# Integrating Multi-Omics with environmental data for precision health: A novel analytic framework and case study on prenatal mercury induced childhood fatty liver disease

**DOI:** 10.1016/j.envint.2024.108930

**Published:** 2024-08-03

**Authors:** Jesse A. Goodrich, Hongxu Wang, Qiran Jia, Nikos Stratakis, Yinqi Zhao, Léa Maitre, Mariona Bustamante, Marina Vafeiadi, Max Aung, Sandra Andrušaitytė, Xavier Basagana, Shohreh F. Farzan, Barbara Heude, Hector Keun, Rob McConnell, Tiffany C. Yang, Alexandros P. Siskos, Jose Urquiza, Damaskini Valvi, Nerea Varo, Line Småstuen Haug, Bente M. Oftedal, Regina Gražulevičienė, Claire Philippat, John Wright, Martine Vrijheid, Leda Chatzi, David V. Conti

**Affiliations:** a Department of Population and Public Health Sciences, University of Southern California, Los Angeles, CA, United States; b Barcelona Institute for Global Health (ISGlobal), Barcelona, Spain; c Universitat Pompeu Fabra (UPF), Barcelona, Spain; d CIBER Epidemiología y Salud Pública (CIBERESP), Spain; e Department of Social Medicine Faculty of Medicine, University of Crete, Heraklion, Greece; f Department of Environmental Sciences, Vytauto Didžiojo Universitetas, Kaunas, Lithuania; g Université de Paris Cité, Institut National de la Santé et de la Recherche Médicale (INSERM), National Research Institute for Agriculture, Food and Environment, Centre of Research in Epidemiology and Statistics, Paris, France; h Department of Surgery & Cancer and Department of Metabolism Digestion & Reproduction Imperial College London, London, United Kingdom; i Bradford Institute for Health Research, Bradford Teaching Hospitals NHS Foundation Trust, Bradford, United Kingdom; j Department of Environmental Medicine and Public Health, Icahn School of Medicine at Mount Sinai, New York, NY, United States; k Laboratory of Biochemistry, University Clinic of Navarra, Pamplona, Spain; l Norwegian Institute of Public Health, Oslo, Norway; m University Grenoble Alpes, Institut National de la Santé et de la Recherche Médicale (INSERM) U 1209, CNRS UMR 5309, Team of Environmental Epidemiology Applied to Development and Respiratory Health, Institute for Advanced Biosciences, 38000 Grenoble, France

**Keywords:** Precision health, Multiomics, Epigenetics, Prenatal exposures, Bioinformatics, Biomarkers

## Abstract

**Background:**

Precision Health aims to revolutionize disease prevention by leveraging information across multiple omic datasets (multi-omics). However, existing methods generally do not consider personalized environmental risk factors (e.g., environmental pollutants).

**Objective:**

To develop and apply a precision health framework which combines multiomic integration (including early, intermediate, and late integration, representing sequential stages at which omics layers are combined for modeling) with mediation approaches (including high-dimensional mediation to identify biomarkers, mediation with latent factors to identify pathways, and integrated/quasi-mediation to identify high-risk subpopulations) to identify novel biomarkers of prenatal mercury induced metabolic dysfunction-associated fatty liver disease (MAFLD), elucidate molecular pathways linking prenatal mercury with MAFLD in children, and identify high-risk children based on integrated exposure and multiomics data.

**Methods:**

This prospective cohort study used data from 420 mother–child pairs from the Human Early Life Exposome (HELIX) project. Mercury concentrations were determined in maternal or cord blood from pregnancy. Cytokeratin 18 (CK-18; a MAFLD biomarker) and five omics layers (DNA Methylation, gene transcription, microRNA, proteins, and metabolites) were measured in blood in childhood (age 6–10 years).

**Results:**

Each standard deviation increase in prenatal mercury was associated with a 0.11 [95% confidence interval: 0.02–0.21] standard deviation increase in CK-18. High dimensional mediation analysis identified 10 biomarkers linking prenatal mercury and CK-18, including six CpG sites and four transcripts. Mediation with latent factors identified molecular pathways linking mercury and MAFLD, including altered cytokine signaling and hepatic stellate cell activation. Integrated/quasi-mediation identified high risk subgroups of children based on unique combinations of exposure levels, omics profiles (driven by epigenetic markers), and MAFLD.

**Conclusions:**

Prenatal mercury exposure is associated with elevated liver enzymes in childhood, likely through alterations in DNA methylation and gene expression. Our analytic framework can be applied across many different fields and serve as a resource to help guide future precision health investigations.

## 1. Introduction

Precision environmental health is an emerging field that aims to integrate environmental and biological factors to identify molecular signatures of environmental exposures, understand mechanisms of environmental disease, and identify high-risk individuals to prevent environment-related diseases (Baccarelli et al., 2023). One of the key biological theories of precision environmental health is that environmental risk factors alter molecular intermediates such as DNA methylation, gene expression, microRNA transcription, and protein and endogenous metabolite levels, leading to disease. Advances in high throughput technologies enable the collective measurement of many molecular intermediates, referred to as omic layers, in a single individual. Although many methods can integrate omic layers to understand disease endophenotypes (Cantini et al., 2021; Gligorijevic and Przulj, 2015), data analysis approaches that integrate environmental factors with multiple omic layers are lacking (Olivier et al., 2019). The absence of a unifying analytic framework is a major barrier to precision environmental health because environmental factors are significant preventable contributors to disease phenotypes.

Identifying molecular mediators and biological pathways linking environmental factors with diseases is critically important for understanding how the environment impacts human health. For example, exposure to mercury, a toxic and ubiquitous heavy metal of global concern (Basu et al., 2018), can impact a broad range of molecular mechanisms, including altering gene regulation (Crespo-Lopez et al., 2009) and increasing oxidative stress (Crespo-Lopez et al., 2009). These molecular changes can increase the risk of various diseases. We have previously shown that prenatal mercury exposure can increase the risk of metabolic dysfunction-associated fatty liver disease (MAFLD) in children (Stratakis et al., Sep 2021; Frediani et al., 2018), an increasingly common childhood disease that increases the risk of severe health outcomes later in life (Welsh et al., 2013; Stefan et al., 2019; Anderson et al., 2015; Pais, et al., 2016; Selvakumar et al., 2017; Adams et al., 2017). Given the breadth of molecular mechanisms that may underlie the relationship between mercury exposure and MAFLD, data on a single omics layer cannot provide a complete picture of how mercury impacts MAFLD risk. Integrating data on mercury exposure with multiple omic layers in an integrated framework has the potential to: (1) identify surrogate multiomic biomarkers of prenatal mercury-related MAFLD years after the exposure; (2) improve the mechanistic understanding of how mercury causes MAFLD; and (3) identify children with the highest risk of mercury-related MAFLD based on individual biological factors. Each of these three goals is a key component of precision environmental health.

Current methods for analyzing multiple omic layers include joint clustering or dimensionality reduction methods (e.g., JIVE, joint matrix/tensor factorization, correlated clustering consensus clustering; Gabasova et al., 2017; Jovanovski and Kocarev, 2019; Kirk et al., 2012; Lock et al., 2013; Luo et al., 2017; Min et al., 2018; Mo et al., 2013; Shen et al., 2009) and network and Bayesian network analysis (e.g., XMWAS; Friedman, 2003; Heckerman, 1998; Liu et al., 2012; Uppal et al., 2018). However, these methods are not designed to examine the indirect effect of environmental factors on disease risk through molecular intermediates in a mediation framework, and instead examine the exposure-omics/omics-outcome associations individually. Several other methods can perform mediation with a single omics dataset, including dimension reduction/mediator screening (Huang and Pan, 2016; Gao et al., 2019), methods accounting for the composite nature of the null (Huang, 2019a,b; Dai et al., 2022; Liu et al., 2022), and methods based on penalized or Bayesian regression (Zhang et al., 2016; Zhao and Luo, 2022; Song et al., 2020; Zeng et al., 2021; Crouse et al., 2022). These high dimensional mediation methods treat all potential mediators in the model equally and therefore do not fully account for the differences in dimensionality, correlation structures, variance, and measurement error between different omics layers. To date, a comprehensive framework for combining existing analytic approaches for integrating multiple omics with existing methods for high dimensional mediation has not been developed.

**Fig. 1.**
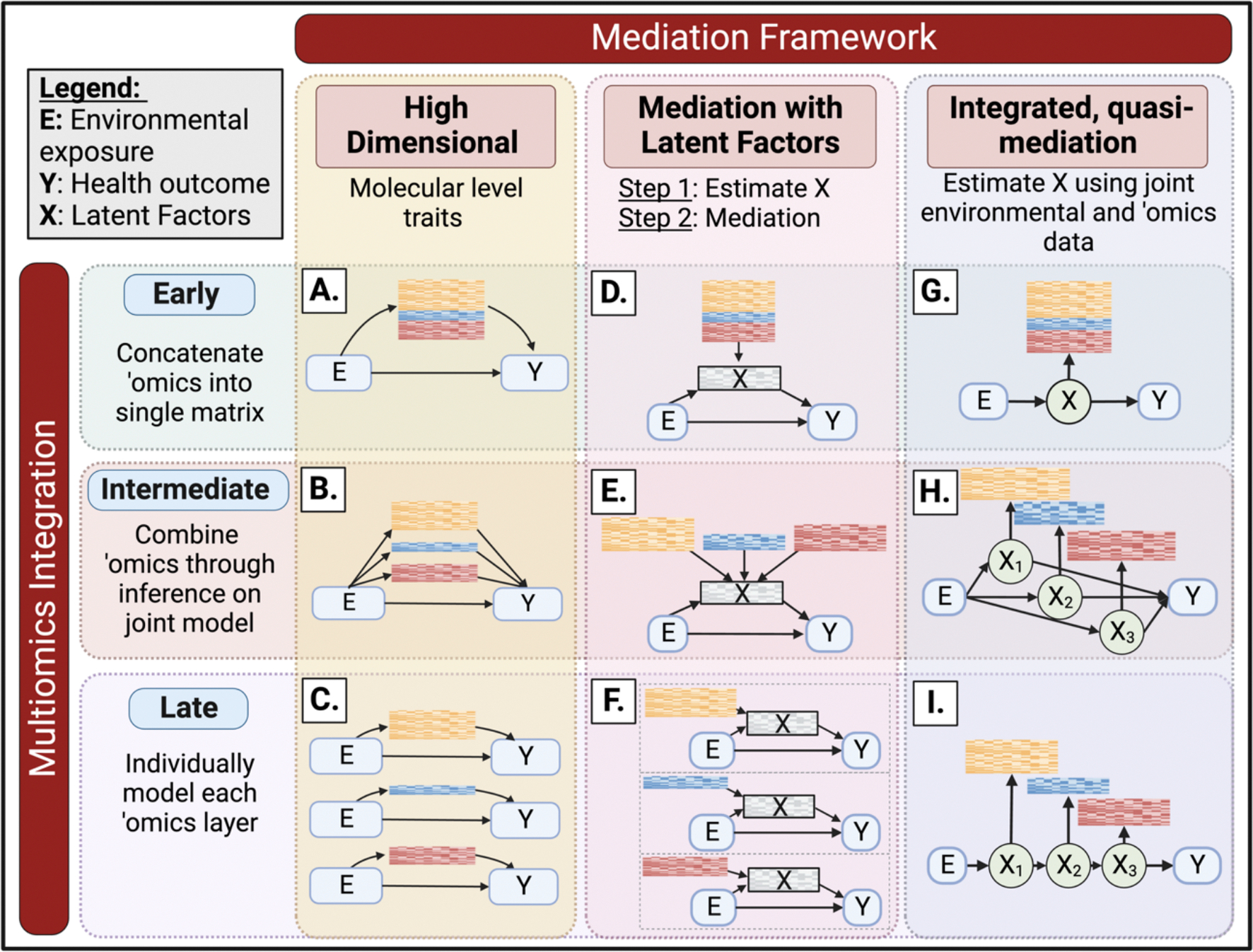
Conceptual diagram illustrating the analytic framework for mediation analysis with multiple omic layers.

To address this gap, we developed a conceptual framework based primarily on existing methods to integrate environmental exposures with multiple omics layers by combining multi-omic data integration methods with mediation analysis (Fig. 1). Our framework defines three approaches for precision environmental health using multi-omic mediation. First, to identify surrogate multi-omic biomarkers of environment-associated disease years after the environmental exposure, we performed a high-dimensional multivariate mediation analysis (Fig. 1, column 1). Second, to identify altered multi-omic pathways that inform on complex molecular mechanisms underlying environment-associated disease, we performed mediation with latent factors (Fig. 1, column 2). Finally, to identify groups of individuals at the highest risk of disease, we estimated latent unknown clusters by jointly analyzing environmental exposures and multi-omic data in a quasi-mediation framework that assumes the entire effect of the exposure on the disease is through molecular intermediates (Fig. 1, column 3).

**Fig. 2.**
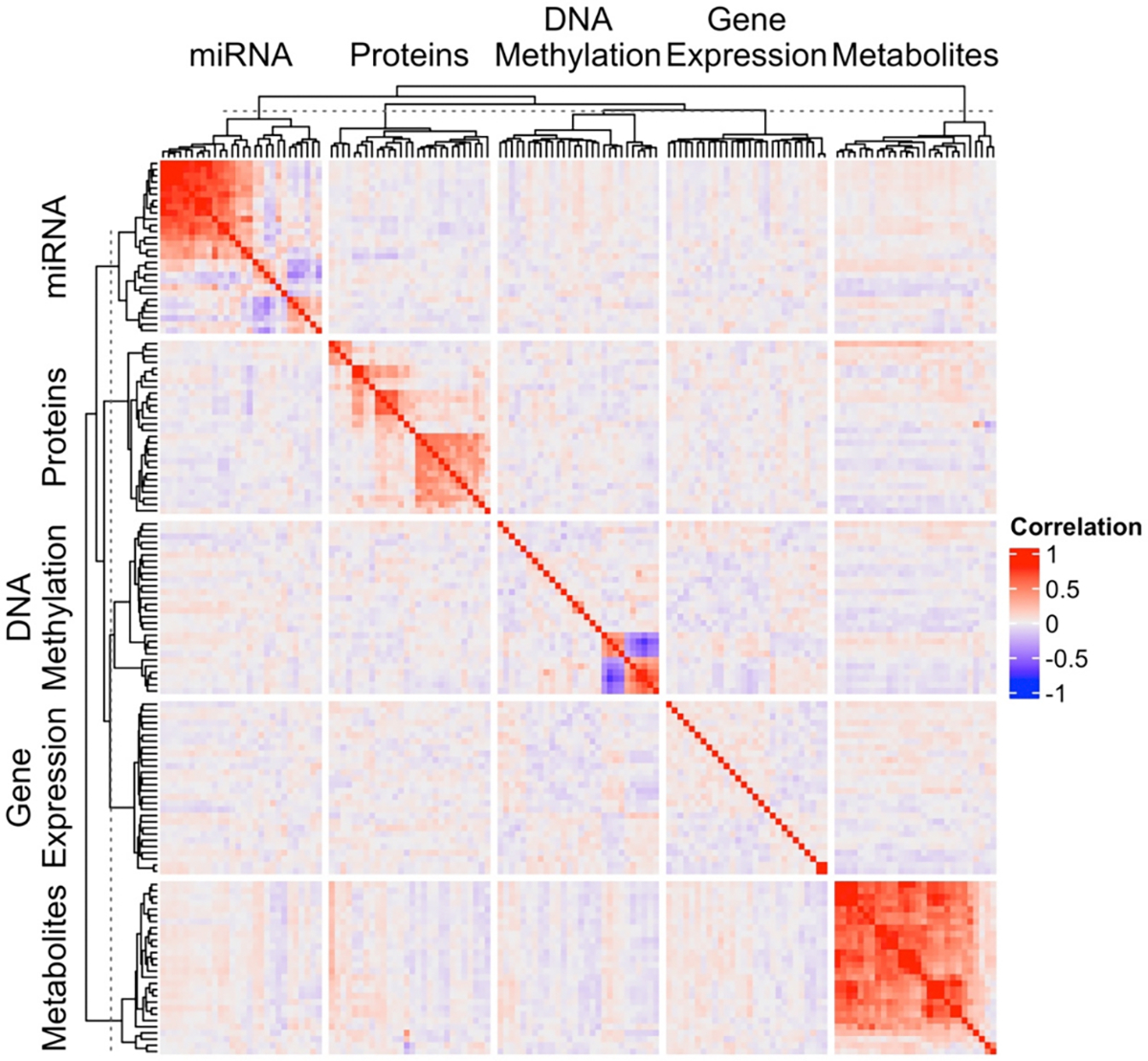
Heatmap illustrating the correlation of molecular features within and between different omics layers. The top 28 features per omic layer were selected for inclusion in the heatmap using a modified version of sure independence screening with prenatal mercury and childhood CK-18.

Across these three mediation approaches, we used three analytic approaches for integrating multi-omic data. The three multi-omic integration approaches include *early integration* (Fig. 1, row 1), in which all omics data matrices are concatenated into a single matrix prior to downstream inferential models; *intermediate integration* (Fig. 1, row 2), in which omics data matrices are combined through inference on a joint model; and *late integration* (Fig. 1, row 3), in which independent inferential models are built for each omics dataset separately, and then single omics features are aggregated collectively in systems-level pathway analyses. These three multi-omic integration approaches account for different aspects of the correlation structures within and between molecular features from multi-omic datasets, which is useful to consider when there are differing correlation structures within and between omics layers (Fig. 2).

Our aim was to develop a resource for researchers by demonstrating how different approaches for mediation with multiple omic layers can be used to address key aspects of precision environmental health. This framework was designed to incorporate continuous or dichotomous outcomes and allows for incorporation of existing multi-omic analysis methodologies. To illustrate how each component of this framework can be used, we applied each approach to data from the Human Early Life Exposome (HELIX) project, which has data on prenatal mercury exposure, childhood multi-omic profiles (DNA methylation, gene expression, micro RNA expression, proteins, and endogenous metabolites), and childhood MAFLD (Haug et al., 2018; Vrijheid et al., 2014).

## 2. Methods

### 2.1. Study design

This prospective cohort study included data from 420 mother–child pairs from the Human Early Life Exposome (HELIX) project (Vrijheid et al., 2014). Details of the HELIX cohort have been published elsewhere (Haug et al., 2018; Vrijheid et al., 2014), but briefly, the HELIX cohort included mother–child pairs from six established and ongoing European population based cohorts, including the Born in Bradford (BiB) study in the UK (Cantini et al., 2021), the Étude des Déterminants pré et postnatals du développement et de la santé de l’Enfant (EDEN) study in France (Gligorijevic and Przulj, 2015), the INfancia y Medio Ambiente (INMA) cohort in Spain (Olivier et al., 2019), the Kaunus cohort (KANC) in Lithuania (Basu et al., 2018), the Norwegian Mother, Father and Child Cohort Study (MoBa) (Crespo-Lopez et al., 2009), and the RHEA Mother Child Cohort study in Crete, Greece (Stratakis et al., 2021). Due to data sharing and privacy regulations, only data from BiB, KANC, RHEA, and INMA were included in the current study.

For the HELIX cohort, pregnant women were recruited and maternal blood samples were collected to determine blood mercury concentrations. For RHEA, BIB, and KANC, whole blood collections occurred during pregnancy (Mean ± SD: 14.1 ± 3.7, 26.6 ± 1.4, and 39.4 ± 1.3 weeks, respectively). In INMA, whole blood during pregnancy was not available, so cord blood was collected at time of delivery. Postnatally, children from the HELIX subcohort underwent a clinical visit between 2013–2016 using standardized protocols across the six cohorts. Average age of children during the clinical visit was 6.6 ± 0.3 years in RHEA, 6.6 ± 0.2 years in BIB, 6.4 ± 0.5 years in KANC, and 8.8 ± 0.5 years in INMA. Clinical visits included detailed questionnaires, a clinical examination, and collection of biospecimens for the characterization of liver enzymes and molecular profiles. At the end of the clinical visit, approximately 18uL of blood was collected after a median fasting time of 3.3 h (5th–95th percentile: 2.2–5.9 h) for determining liver enzymes and molecular phenotypes (Maitre et al., 2018). Blood samples were processed with methods using standardized protocols across all cohorts (Maitre et al., 2018).

All participants (mothers and children) provided written informed consent/assent before participation. Study procedures across all cohorts were conducted based on the Declaration of Helsinki guidelines. Approval for the HELIX project was obtained at each site by local ethics committees. The University of Southern California Institutional Review Board approved the current study.

### 2.2. Maternal blood mercury concentrations

For the BIB, KANC, and RHEA cohorts, measurements of total mercury in whole blood were measured using inductively coupled plasma-mass spectrometry, as described previously (Haug et al., 2018). Whole blood samples from pregnancy were not available in the INMA cohort, so for this cohort, total mercury in cord whole blood samples was measured using thermal decomposition, amalgamation, and atomic absorption spectrometry. To obtain comparable total mercury levels in INMA to those from maternal whole blood in the BIB, KANC, and RHEA cohorts, total mercury was divided by 1.7 (Stern and Smith, 2003). These measurements of total mercury represent both organic (including methylmercury) and inorganic species of mercury.

### 2.3. Liver enzymes in childhood

Levels of alanine aminotransferase (ALT), aspartate aminotransferase (AST), gamma-glutamyl transferase (GGT), and cytokeratin 18 (CK-18) were quantified in serum at the Biochemistry Laboratory of the Clínica Universidad de Navarra (Spaine). The primary outcome of this study was CK-18, as it has been shown to be a strong predictor of MAFLD in children (Vos et al., 2008).

### 2.4. Molecular phenotypes

Five omic assays were performed on child biospecimens, as described previously (Maitre et al., 2022). DNA methylation was quantified in buffy coat using the 450 K array (Illumina), gene expression in whole blood were determined using the HTA V.2.0 array (Affymetrix), levels of miRNA in whole blood were determined using the SurePrint Human miRNA rel 21 assay (Agilent), levels of proteins were determined using the cytokines 30-plex, apoliprotein 5-plex and adipokine 15-plex assays (Luminex), and levels of serum metabolites were determined using the AbsoluteIDQ p180 kit (Biocrates). Prior to measurement, samples were randomized and blocked by cohort to ensure that each cohort was represented in each batch (Maitre et al., 2022). Data processing and quality control was performed as described previously (Maitre et al., 2022). Following quality control analysis, 386,519 CpG sites, 58,254 transcript clusters, 1,117 miRNA, 36 proteins, and 177 serum metabolites were included in analysis.

### 2.5. Data analysis

We used linear regression to examine the association between prenatal mercury levels and childhood CK-18. To achieve a parsimonious model that minimized the risk of confounding while avoiding overadjustment and potential bias, all analyses were adjusted for a minimal set of covariates, including HELIX cohort, child sex, child age, and maternal fish intake, categorized as <2 times per week, 2–4 times per week, and >4 times per week. To arrive at this minimum set of adjustment variables, we took the following approach. First, we identified a set of key confounders, which included HELIX cohort, maternal fish consumption, child sex, and child age. These variables were adjusted for in all analyses, as they could either confound the exposure outcome association (HELIX cohort, maternal fish consumption) or the mediator outcome association (child sex, child age). In addition to these variables, we examined an extended set of potential covariates for the exposure outcome associations based on the directed acyclic graph (DAG) presented by Stratakis et al., 2021 (Stratakis et al., 2021). These covariates included maternal age, maternal education, parity, maternal BMI, maternal smoking status, and child BMI. We tested and retained covariates in the models using a forward selection approach, starting with the minimal model containing the exposure and the four key adjustment variables. For this forward selection process, covariates were retained in the model if they altered the effect estimate for the exposure outcome association more than 10% (Lee, 2014). None of these additional confounders met this criteria, and thus, no additional confounders were included in the models.

We performed two additional sensitivity analyses for the associations between prenatal mercury levels and childhood CK-18. First, we performed a fixed effects meta-analysis to examine heterogeneity between cohorts, using the R package *meta* (Schwarzer et al., 2015). Second, to examine the robustness of our findings, we examined the potential impact of unknown/unobserved confounders on our results by calculating the robustness value. The robustness value represents the minimum strength of association (as a percent of variance explained) between the unknown confounder with the residual variance in the exposure and the residual variance in the outcome which is needed to reduce the observed effect to zero (Cinelli and Hazlett, 2020).

In all mediation analyses, we included the same covariates as the exposure-outcome analysis for both the exposure-mediator and mediator-outcome associations. To minimize the impact of cohort on the mediation results in our study, prenatal mercury and childhood CK-18 were scaled within each cohort to a mean of zero and standard deviation of one. This standardization helped account for the potential for differences in cohorts to confound the associations between the exposure and the multiomics features as well as the multiomics features and the outcome. Before analysis, molecular features from all omic layers were adjusted for study cohort, child age, and child sex and scaled to a mean of zero and a standard deviation of one by calculating the studentized residuals from the linear regression of each omics feature as the dependent variable and study cohort, child age, and child sex as the independent variables.

Since some of the methods including HIMA (Zhang et al., 2016) and LUCID (Peng et al., 2020) employ a pre-selection procedure to reduce the number of omics features before analysis, we performed a preselection procedure prior to all analyses to maintain consistency across all methods. This pre-selection procedure was performed using a modified meet in the middle approach to identify the most important omic features potentially linking the exposure and the outcome (Chadeau-Hyam et al., 2011), and a sure independence screening approach to determine the number of features to be included in the final analysis (Fan and Lv, 2008). Overall, this selection approach is similar to that implemented in HIMA (Zhang et al., 2016). First, to identify and rank individual omics features within each omic layer, we used a modified meet in the middle approach. We performed individual regressions between the exposure and individual omic feature adjusting for covariates, and regressions of each individual omic feature with the outcome, adjusting for covariates and the exposure (Chadeau-Hyam et al., 2011). Since this procedure provides two estimates (the exposure-omic effect estimate α and the omic feature-outcome effect estimate β), it is challenging to rank the top omic features. Thus, we additionally calculated the product of the coefficients (α*β) to estimate the potential mediating variable effect. This was used to identify the top 28 omic features per omic layer, by selecting those with the largest product of the coefficients. The 28 features per layer was determined using the sure independent screening approach which is based on the total study sample size, n. Specifically, the total number of omic features included in final analysis was determined using [2n/log(n)] (Zhang et al., 2016). The resulting number, (2*140)/log(140) = 139.0, was rounded up to the closest multiple of five. Finally, this number was divided across the five omics layers evenly, resulting in 28 omics features per layer and 140 omics features overall.

### 2.6. High dimensional mediation analysis with multiple omics (Fig. 1, column 1)

To illustrate high dimensional mediation analysis with multiple omics, we used two different methods. For early and late integration, we used High Dimensional Mediation Analysis (HIMA), a penalization-based mediation method implemented in the R package HIMA (Zhang et al., 2016). For intermediate integration, we employed a novel two-step approach that incorporates feature level metadata to inform on feature selection using *xtune* (Zeng et al., 2021), an approach that allows for omic layer specific penalty parameters and, in this example, performs a group-lasso-type shrinkage within each omic dataset. This analysis is similar to HIMA, except that for this method the penalization can vary across each of the omic layers. This analysis was based on the product of coefficients method for mediation and was performed in three steps. First, we performed independent linear regression models for all exposure-mediator associations to get the exposure mediator coefficient. Second, we performed a single group lasso regression for the mediator outcome associations, adjusting for the exposure, using the R package xtune (Zeng et al., 2021; He and Zeng, 2023). This step provided coefficients for each of the mediator outcome associations. We used bootstrapping to obtain the standard error of the coefficients from the group lasso regression for each of the mediator coefficients. Finally, for each omic feature, we calculated the mediation effect and 95% confidence intervals using the distribution of the product method (Williams and MacKinnon, 2008) using the R package RMediation (Tofighi and MacKinnon, 2011).

### 2.7. Mediation with latent factors (Fig. 1, column 2)

Here, we identify these latent factors using dimensionality reduction techniques such that the latent factors describe individual and/or joint variability across the multiple omic layers. For early and late integration, we used principal component analysis (PCA) as a dimensionality reduction step and selected the top *i* principal components which explained >80% of the variance. For intermediate integration, we used a PCA based multi-omic integration method which decomposes the variance across multiple omic layers into joint and individual variance components using the R package JIVE (Lock et al., 2013). For the JIVE algorithm, rank selection for the joint and individual variance components was performed using the permutation method. Following the joint dimensionality reduction step, we used the r package HIMA to examine whether the variance components mediated associations of prenatal mercury exposure with MAFLD.

To examine how individual omics features were associated with variance components that mediated the exposure-outcome association, we calculated the Pearson correlation coefficient and corresponding p-value between each omic feature and each variance component. Using QIAGEN IPA (QIAGEN Inc.), we then performed a pathway analysis on each variance component separately using the p-values for each omic feature as the input. For pathway analysis, we used a p-value threshold of 0.05 within IPA to identify statistically significant features.

### 2.8. Integrated/Quasi-mediation (Fig. 1, column 3)

To illustrate integrated/quasi-mediation, we used three different implementations of a Latent Unknown Clustering method for Integrating multi-omics data (LUCID). The overarching LUCID framework, which was initially developed to integrate a single omics dataset, has been described previously (Peng et al., 2020). Here, we implement three different LUCID models, including the original LUCID model for early integration, LUCID in parallel, an extension to LUCID for performing intermediate integration, and LUCID in serial, an extension to LUCID for performing late integration. For consistency across early, intermediate, and late integration, we a-priori set the number of latent clusters per omic layer equal to two for all analyses and omics layers and we did not perform variable selection on the omics features. For this analysis, we only included the methylome, the transcriptome, and miRNA as the omics datasets in order to improve interpretation and reduce the model complexity. For example, for quasi-mediation with intermediate integration, including all 5 omics layers and estimating two omics specific clusters per layer would lead to 2^5^, or 32, distinct groups of individuals. By including only three omics datasets, the number of groups was reduced to 2^3^, or 8 groups of individuals.

For early integration, we used the original supervised LUCID model implemented in the R package *LUCIDus* to conduct integrated clustering of concatenated omic data conditional on the exposure and the outcome (Peng et al., 2020). In this context, the supervised model uses information on the outcome to assist in defining the latent clusters. LUCID also estimates omics profiles for each latent cluster by calculating the expected value of each omic feature within the corresponding cluster. These omic profiles confer different risks of the outcome and represent each omics feature’s contribution to the exposure-outcome association.

For intermediate integration, we implemented a supervised LUCID model in parallel. LUCID in parallel estimates latent clusters in each omic layer separately while integrating information from the exposure and the outcome by assuming no correlations across different omics layers. LUCID in parallel estimates omics specific clusters which represent differential risks for the outcome within the layer. For each set of latent clusters within each omic layer, the corresponding omics profile was computed to identify the independent contribution of each omic layer to the exposure-outcome association.

For late integration, we implemented LUCID in serial by successively linking multiple single omics LUCID models using the original LUCID model. We linked successive omics layers by using each participant’s posterior inclusion probability (PIP) for latent clusters in the initial LUCID model to be the “exposure” variable for each successive model. We ordered the omic layers in a sequential fashion based on the biological relationships between omic layers. For the first model, we fit an unsupervised original LUCID model using mercury as the exposure and DNA methylation as the omic layer. The PIPs of the non-reference clusters were extracted and used as the input for the exposure for the following unsupervised LUCID model. We iterated this procedure until the last omic layer, for which we used a supervised original LUCID model to conduct integrated clustering while using PIPs from the previous LUCID model and information on the outcome.

### 2.9. Data availability

The summarized results (exposure, omics biomarker, effect, standard error, p value) generated during this study are available in the Supplemental Tables. The raw data supporting the current study are available from the corresponding author on request subject to ethical and legislative review. The “HELIX Data External Data Request Procedures” are available with the data inventory in this website: https://www.projecthelix.eu/data-inventory. The document describes who can apply to the data and how, the timings for approval and the conditions to data access and publication. The simulated data used to demonstrate our framework is available at https://github.com/Goodrich-Lab/EnvirOmix and in Zenodo at https://doi.org/10.5281/zenodo.10067991.

### 2.10. Code availability and software

All analyses were performed using R version 4.3.1. A complete list of the R packages and versions used throughout this analysis are provided in the Supplemental Methods. An interactive version of the code used in this analysis is provided at https://goodrich-lab.github.io/multiomics_book/, and a static version of this code can be accessed at https://doi.org/10.5281/zenodo.10067991.

## 3. Results

### 3.1. Prenatal mercury exposure is associated with risk for MAFLD in childhood

Participant characteristics and prenatal blood mercury concentrations for the 420 mother–child pairs included in the analysis are in Table 1. There was a positive association between prenatal blood mercury levels and childhood CK-18 in children aged 8 years. When adjusting for cohort, sex, child age, and prenatal fish intake, each standard deviation (SD) increase in prenatal mercury was associated with a 0.11 [95% confidence interval: 0.01, 0.21] SD increase in CK-18 (p = 0.02). Individual cohort estimates ranged from 0.03 [−0.15, 0.22] in RHEA to 0.28 [0.03, 0.54] in BIB. The chi-squared test for heterogeneity indicated that there was no statistically significant heterogeneity between studies (χ^2^ = 2.45; p = 0.48; Figure S1). Further, the robustness value of this association was 10.6%, indicating that an unknown or unmeasured confounder would have to explain more than 10.6% of the residual variance in both the exposure and the outcome to reduce the observed association between prenatal mercury and CK-18 to zero. As context, maternal fish intake only explained 2.6% of the variance in maternal mercury levels but explained less than 0.5% of the variance in childhood CK-18 levels.

### 3.2. Biomarkers of mercury-associated hepatotoxicity via high dimensional mediation

Individual molecular features linking prenatal mercury with liver injury can be used as surrogate markers of environment-associated disease risk, even years after the exposure (Fig. 1, Column 1). High dimensional mediation methods use penalization (i.e., lasso or ridge) that select the features with the strongest effects and shrink the estimates from other features towards zero. This potentially reduces model complexity and is ideal for identifying the minimum set of informative biomarkers potentially linking prenatal mercury to liver injury. However, shrinkage approaches that prioritize model simplicity can result in biased estimates. Moreover, when analyzing highly correlated data, approaches aimed to achieve sparsity, for example lasso, will select a single feature among the correlated set and remove all others. Thus, this method is ideal for identifying a minimal set of biomarkers but is less suited for subsequent pathway enrichment analysis.

**Fig. 3.**
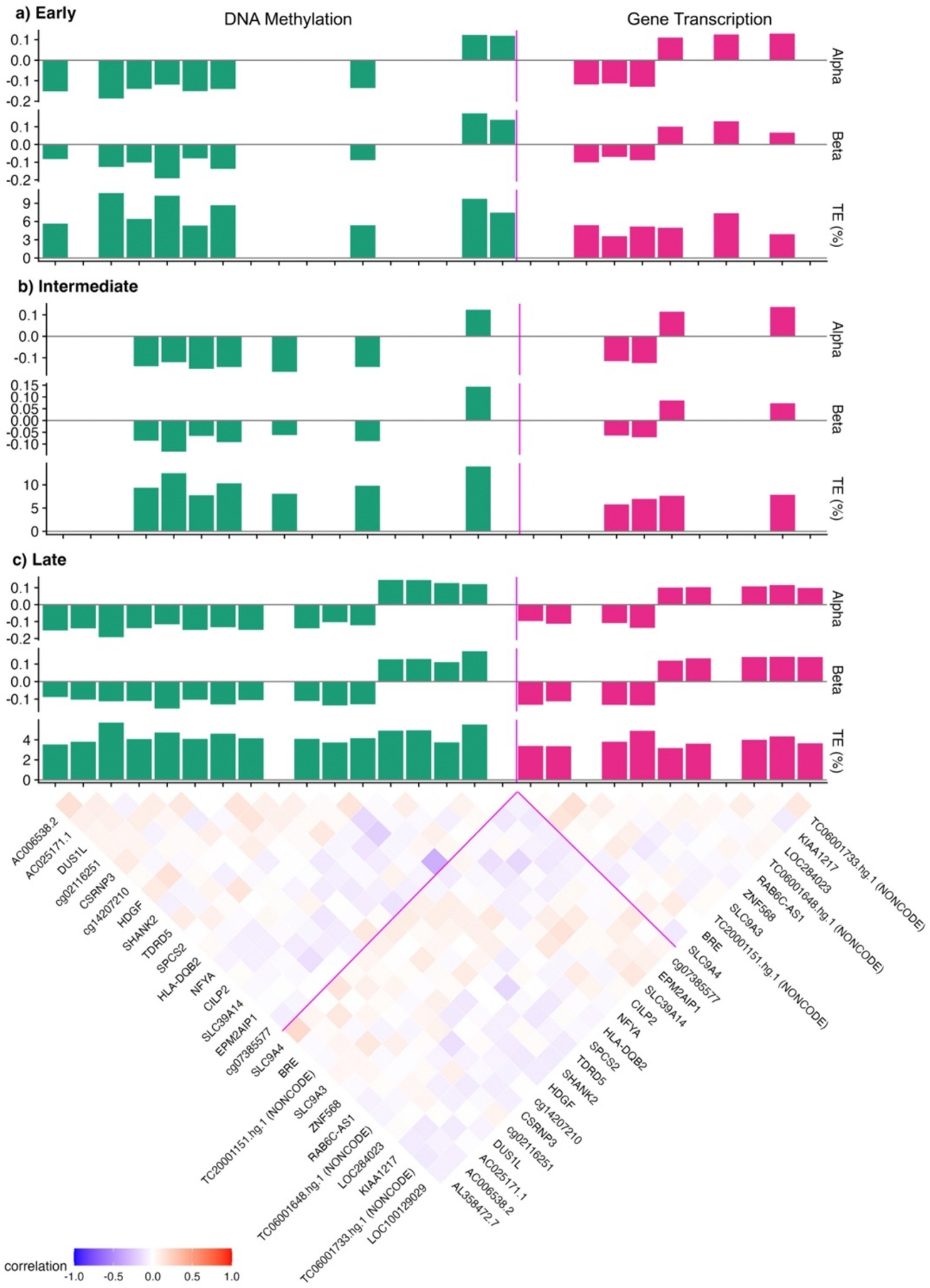
High dimensional mediation analysis with multiple omic layers identifies individual molecular features linking prenatal mercury with childhood liver injury. **Panels A, B, and C** present the results for early integration (described in Fig. 1A), intermediate integration (described in Fig. 1B), and late integration (described in Fig. 1C), respectively, with each column representing a single omics feature. Alpha represents the coefficient estimates of the exposure to the mediator, Beta indicates the coefficient estimates of the mediators to the outcome, and TE (%) represents the percent total effect mediated calculated as alpha*beta/gamma. The triangular heatmap at the bottom of the figure shows the correlation between individual omics features.

High dimensional mediation with early integration (Fig. 1A) identified nine differentially methylated CpG sites and six differentially expressed gene transcript clusters which link prenatal mercury with childhood CK-18 in children (Fig. 3a; Table S1). Across the nine CpG sites, the percent total effect mediated ranged from 5% to 11%, with the highest effect observed for a CpG associated with the *AC025171.1/ZNF131* genes; a one SD increase in mercury was associated with 0.19 decrease of this CpG, while a one-unit increase in this CpG was associated with 0.13 SD decrease in CK-18 in children. Consequently, the percent total effect mediated was 10.68% (Alpha = − 0.19; Beta = − 0.13; %TE = 10.68%) (Table S1). Across the six differentially expressed gene transcripts, the percentage total effect mediated varied between 3% and 8%, with the highest effect observed for an expressed gene transcript cluster associated with the LOC284023 gene. Specifically, a one-SD increase in mercury was associated with a 0.13 SD increase in this transcript, and a one-SD increase in this transcript was associated with a 0.13 SD increase in CK-18 in children; the percent total effect mediated was 7.4% (Alpha = 0.13; Beta = 0.13; %TE = 7.4%) (Table S1). For this approach, combining all omics layers before analysis identified the strongest feature linking environmental and disease across all omics layers without accounting for the differences in underlying correlation structure. Eight of the CpG sites and four of the transcripts were also identified in either intermediate or late integration, while one CpG site and two differentially expressed transcripts were identified only with early integration.

High dimensional mediation with intermediate integration (Fig. 1B) differs from early integration because while the omics layers are analyzed jointly, the feature selection penalization is allowed to vary between different omics layers. This approach accounts for differences in the overall impact between different omics layers. This approach identified seven differentially methylated CpG sites and four differentially expressed gene transcript which link prenatal mercury with childhood CK-18, with percent total effect mediated ranging from 7% to 14% and 5% to 8%, respectively (Fig. 3b; Table S1). The CpG site associated with *EPM2AIP1/MLH1* was observed to have the highest percent total effect mediated among the seven differentially methylated CpG sites (Alpha = 0.12; Beta = 0.14; %TE = 13.9%). Across the four differentially expressed gene transcripts, the highest percent total effect mediated was 7.8% for a small nucleolar RNA (snoRNA) on chromosome 6 (TC06001733.hg.1; Alpha = 0.14; Beta = 0.07; %TE = 7.8%). While 10 of these features overlapped with both early and late integration, intermediate integration identified one differential methylated CpG which was not identified in early or late integration.

High dimensional mediation with late integration (Fig. 1C) differs from the early and intermediate integration in that each omic layer is analyzed individually. Thus, this approach does not condition on features within the other omics layers in the analysis. This approach identified the most omic features across integration approaches, including 15 differentially methylated CpG sites and nine differentially expressed transcript clusters linking prenatal mercury with childhood CK-18 (Fig. 3c; Table S1). While 12 of these features overlapped with features identified in early and intermediation, late integration identified seven additional differentially methylated CpG sites and five differentially expressed transcripts what were not identified in either of the other approaches. Across the 15 CpG sites, the percent total effect mediated ranged from 2% to 7%, with the highest effect observed for a CpG associated with the *EPM2AIP1/MLH1* genes; a one SD increase in mercury was associated with a 0.12 SD increase in this CpG, while a one SD increase in this CpG was associated with 0.19 SD increase in CK-18 in children. Consequently, the percent total effect mediated was 6.3% (Alpha = 0.12; Beta = 0.19; %TE = 6.3%) (Table S1). Across the nine differentially expressed gene transcripts, the percentage total effect mediated varied between 3% and 5%, with the highest being 5.0% for a snoRNA on chromosome 6 (TC06001733.hg.1; Alpha = 0.13; Beta = 0.14; %TE = 5.0%).

Across all three high dimensional mediation approaches, 6 CpG sites and 4 transcript clusters were consistently identified as linking prenatal mercury and childhood MAFLD. The strongest association was observed with a hypermethylation at a CpG site associated with the *EPM2AIP1/MLH1* genes, which was identified across early, intermediate, and late integration. Due to the nature of the penalization, the correlations between the selected omics features within and between omic datasets were relatively low (Fig. 3d). The exception to this is for late integration, which is more likely to select correlated features between different omics layers.

### 3.3. Mediation analysis with latent factors informs on molecular pathways of mercury induced hepatotoxicity

Uncovering exposure-disease mechanisms is an essential component of precision environmental health. It allows for stronger inference on disease-outcome associations and may help identify therapeutic targets for precision health. While individual features can provide some insight into mechanisms, identifying multi-omic pathways that link exposure and outcomes can provide a more comprehensive understanding of the mechanisms linking exposure and disease. To identify altered pathways, we define a two-step approach to first identify latent factors, then characterize specific components of the multi-omics data, followed by mediation analysis that incorporates these latent factors as mediators (Fig. 1, column 2). These methods generally result in unbiased effect estimates across all multi-omic features in the dataset, making this approach ideal for performing subsequent pathway analysis.

**Fig. 4.**
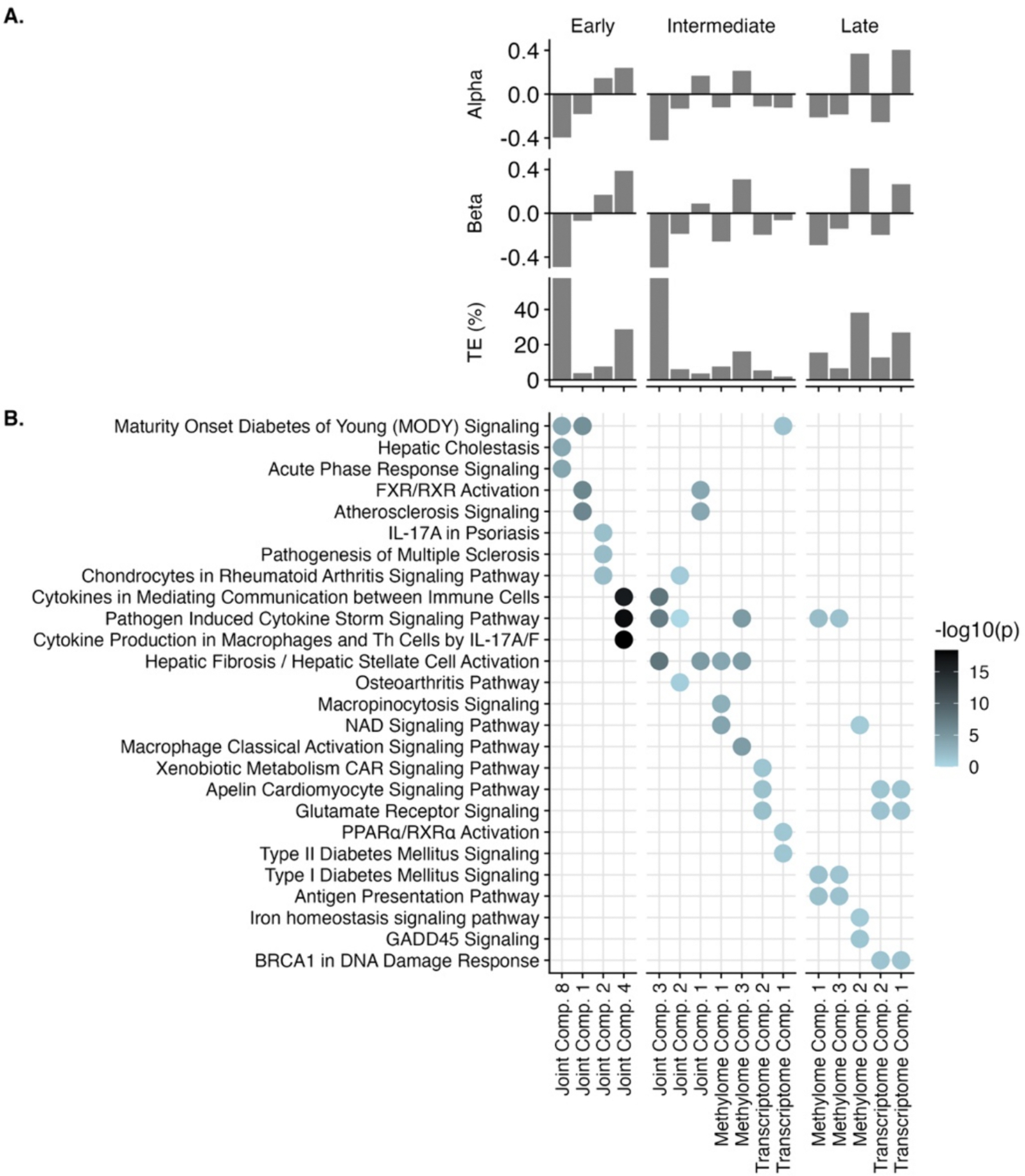
Mediation analysis with latent factors identifies alterations in critical molecular pathways linking prenatal mercury exposure with childhood CK-18 (Fig. 1, Column 2). **Panel A** illustrates the mediation effects for each of the three different omics integration approaches (early, intermediate, and late), where alpha is the coefficient estimate of the exposure to the mediator, beta is the coefficient estimate of the mediators to the outcome (PCs to childhood liver enzymes), and % TE indicates the percent of total effect mediated, calculated as alpha*beta/total effect and scaled to 100%. Each column represents either a joint variance component (describing common variance across all omics) or an individual variance component (describing variance specific to that omic layer). **Panel B** illustrates the top three molecular pathways associated with each individual or joint variance component.

**Fig. 5.**
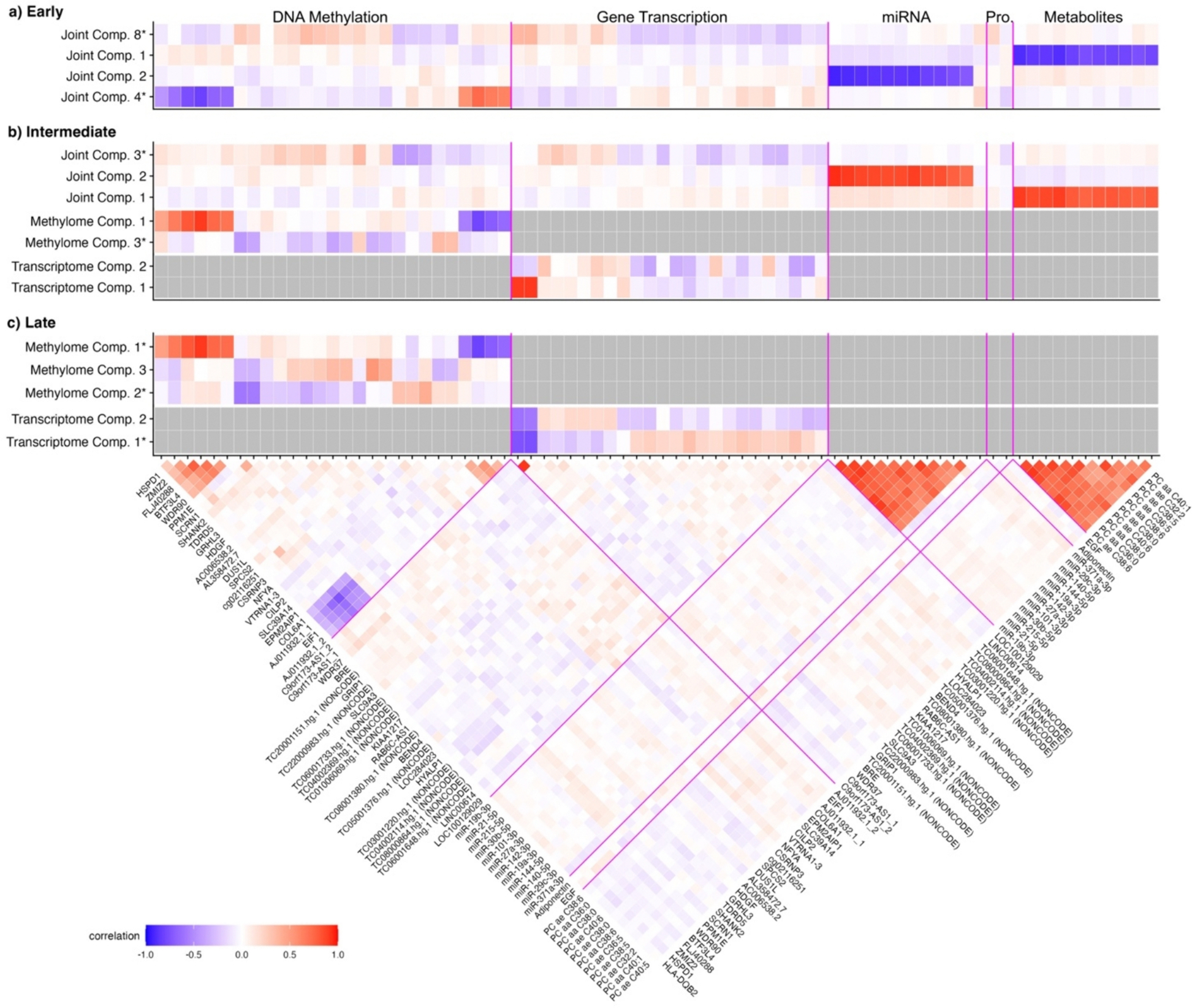
Associations of individual omic features with joint and individual variance components mediating the association of prenatal mercury exposure with childhood MAFLD were determined using mediation analysis with latent factors (Fig. 1, Column 2). **Panels A-C** represent the correlation of each omic feature with each variance component calculated using three different omics integration approaches. For omics specific components (i.e., methylome components and transcriptome components), grey colors indicate omics features not associated with that specific omic layer. The triangular heatmap at the bottom of the figure shows the correlation between individual omics features. Vertical magenta lines in panels A-C separate features from different omics layers. * indicates joint components which mediated greater than 15% of the total effect. (For interpretation of the references to colour in this figure legend, the reader is referred to the web version of this article.)

Determining latent factors with early integration (Fig. 1D) implemented using PCA identified 55 joint components that together explained 80% of the variation across all omics layers (Table S2). Using high dimensional mediation analysis, four of the joint components mediated associations of prenatal mercury and childhood CK-18 at a false discovery rate-corrected (FDR) p < 0.05 (Fig. 4a). The component with the largest mediation effect (joint component eight) was associated with hepatic cholestasis and signaling related to acute phase response and linked to maturity onset diabetes of young (MODY; Fig. 4b); this component explained 59.8% of the total mediation effect. These pathways were driven by lower levels of several gene transcripts and differential methylation of several protein coding genes (Fig. 5a). In contrast to joint components 1 and 2 which were driven primarily by alterations in metabolites and miRNA, joint component eight was driven by alterations in DNA methylation and gene transcription. The top two CpG sites associated with joint component eight were both associated with genes *C9orf173-AS1,* with Pearson correlation coefficients with the joint component of 0.37 and 0.36, respectively. The top two gene transcripts associated with joint component eight were linked to the genes *HDGF*/*PRCC* and *AC006538.2*/*SLC39A3,* with Pearson correlation coefficients of 0.34 and 0.29, respectively (Table S2).

Determining latent factors with intermediate integration was performed using JIVE (Fig. 1E). This approach identified the optimal number of latent factors to be three joint components and between three to six individual components (methylome: three components; transcriptome: five components; miRNA: four components; proteome: five components; metabolome: six components; Table S2). With high-dimensional mediation, all three joint components, two methylome specific components, and two transcriptome specific components significantly linked prenatal mercury and MAFLD at an FDR p < 0.05 (Fig. 4a). The component with the strongest total mediation effect was joint component three (%TE mediated: 59.2%). This component was associated with differential methylation of several protein coding genes, including *NFYA* (r = 0.42), *VTRNA1–3* (r = 0.42), *SPCS2* (r = − 0.35), and *CILP2* (r = 0.34), and altered levels of several gene transcripts, including *BRE* (r = − 0.28) and *KIAA1217* (r = 0.27; Fig. 5b; Table S2). Pathway analysis on these features determined that joint component three was associated with altered cytokine signaling and hepatic stellate cell activation/hepatic fibrosis (Fig. 4b).

Determining latent factors using late integration (Fig. 1F) implemented using PCA identified between seven to twenty individual components for each omic layer that each explained 80% of the variation in the respective omic layer (methylome: 17 components; transcriptome: 20 components; miRNA: 10 components; proteome: 14 components; metabolome: 7 components; Table S2). Using high dimensional mediation, three methylome specific components and two transcriptome specific components significantly linked prenatal mercury and risk of MAFLD at an FDR p < 0.05 (Fig. 4a). The component with the strongest effect was methylome component 2, which was associated with lower DNA methylation at the protein coding genes *PPM1E* (r = − 0.59) and *SCRN1* (r = − 0.58), among others (Fig. 5c; Table S2), and was defined by altered cytokine storm signaling, iron homeostasis signaling, and GADD45 signaling (Fig. 4b).

Since mediation analysis with latent factors does not specifically perform feature selection, the features that contributed substantially to the components in each analysis were more correlated than those identified from high dimensional mediation (Fig. 5). These correlated metabolites and miRNA drove several of the significant components. Since correlation among molecular features suggests common molecular regulatory mechanisms, each component may be indicative of altered regulation related to a shared biological mechanism.

### 3.4. Integrated/quasi-mediation analysis of mercury and multiple omics identifies high risk subpopulations

Identifying subgroups of children at high risk of disease using information on environmental exposures and multi-omic profiles is a key goal of precision environmental health, as it allows for better prediction, prevention, and personalized intervention strategies. By identifying susceptible groups early, interventions can be tailored to each individual’s unique environmental and biological context to reduce the incidence of environment associated disease. To identify subgroups, we define a quasi-mediation approach using LUCID, where information on environmental factors and information on multiple omic layers is analyzed jointly in a single unified analysis (Fig. 1, column 3). This approach differs from high dimensional multi-omic mediation analysis and mediation analysis with latent factors in that it does not formally estimate the direct and indirect effects. Instead, this integrated analysis assumes the entire environmental effect is through latent factors that provide insight into relevant subgroups (as determined via the exposure and multiple omic layers) that capture clinically relevant profiles associated with MAFLD risk.

Quasi-mediation analysis with early integration (Fig. 1G) identified two subgroups of participants with different exposure levels, omics profiles, and childhood CK-18 (Figure S2; Table S3). Each SD increase in prenatal mercury was associated with a non-significant 2.39 times higher odds of inclusion in a high-risk subgroup characterized by omics profile 1 (95% CI: 1.54–3.82; p = 0.00013). Omics profile 1 was defined by differential DNA methylation, differential gene transcription, and lower miRNA levels. Individuals in the high-risk subgroup exhibited 3.36 SD higher CK-18 compared to individuals in the low-risk subgroup (95% CI: 3.00–3.72; p < 0.00001).

**Fig. 6.**
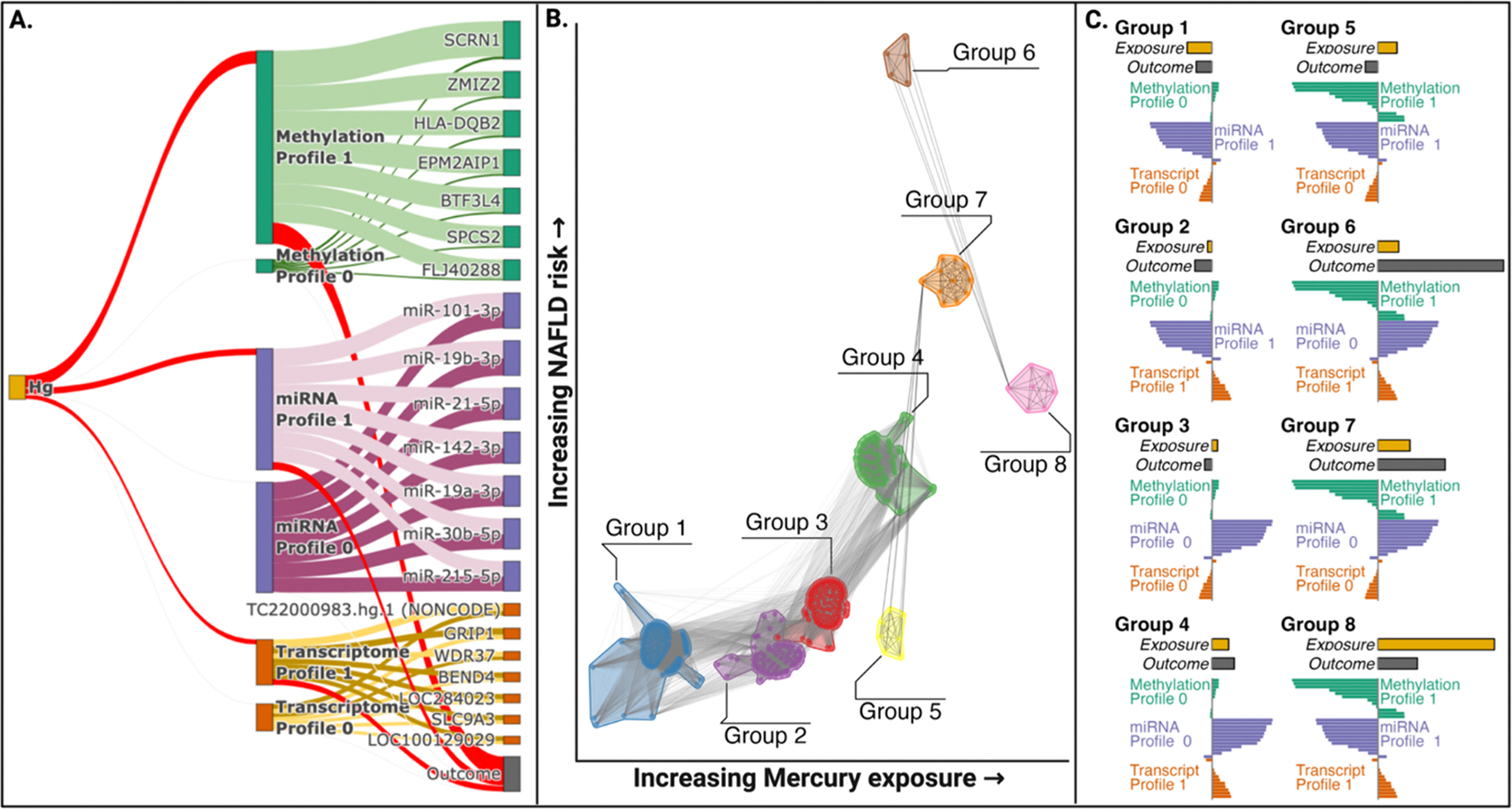
Quasi-mediation analysis with intermediate integration identifies eight distinct groups of children at high risk of MAFLD based on prenatal mercury exposure and distinct omics profiles (Fig. 1, Column 3). **Panel A** shows the associations of prenatal mercury exposure (on left) with different omics profiles (in the middle). The red line connecting mercury exposure with each of the omics profiles indicates positive associations between prenatal mercury exposure, with the width of the lines being proportional to the magnitude of the association. The red line connecting each of the omics profiles with the outcome indicates that these omics profiles are associated with higher risk of liver injury in childhood. The dark green, dark purple, and dark gold lines indicate positive associations between the omic profile and the omic feature. In contrast, the light green, light purple, and light gold lines indicate negative associations. **Panel B** illustrates the eight groups of individuals with unique exposure, omics, and outcome profiles. Points indicate individuals and lines connect individuals with similar exposure, omic, and outcome profiles. Groups are positioned in order of increasing exposure and childhood CK-18. **Panel C** shows the exposure levels, childhood CK-18, and omics profiles for each of the eight groups in panel B. For example, group 1 has low exposure and low childhood CK-18 and is characterized by methylation profile 0, transcriptome profile 0, and miRNA profile 1. In contrast, group 8 has high exposure, moderate to high risk of MAFLD, and methylation profile 1, transcriptome profile 1, and miRNA profile 1. (For interpretation of the references to colour in this figure legend, the reader is referred to the web version of this article.)

Quasi-mediation analysis with intermediate integration (Fig. 1H) identified two omic profiles for each omic layer, resulting in six omic profiles characterized by differential exposure levels and childhood CK-18 (Fig. 6A; Table S3). Methylation profile 1 had the strongest association with mercury exposure and childhood CK-18 compared to omic profiles from miRNA and gene transcription. Each SD increase in prenatal mercury was associated with 2.16-times higher odds of being in high-risk methylation profile 1 (95% CI: 1.48–3.13; p = 0.00006). Individuals in this high-risk subgroup exhibited 1.18 SD higher CK-18 compared to individuals in the low-risk methylation profile 0 (95% CI: 0.82–1.54; p < 0.00001). For miRNA, each SD increase in mercury resulted in 1.23 times higher odds of inclusion in the high risk miRNA profile 1 (95% CI: 1.02–1.48; p = 0.034); miRNA profile 1 was associated with a 0.25 SD higher CK-18 compared to the low-risk miRNA Profile 0 (95% CI: 0.06–0.43; p = 0.008). Finally, each SD increase in prenatal mercury was associated with a 1.22-times higher odds of being in the high-risk gene transcription profile 1 (95% CI: 1.01–1.47; p = 0.04); childhood CK-18 levels were 0.21 SD higher in this high risk profile than in the low-risk gene transcription profile 0 (95% CI: 0.02–0.39; p = 0.027). Based on the six omics specific profiles, we identified eight subgroups of children (Fig. 6B), each exhibiting a unique combination of exposure levels, omics profiles, and childhood CK-18 (Fig. 6C). This allowed us to identify individuals at the highest risk of prenatal mercury induced MAFLD. For example, group 8 had significantly elevated exposure levels and a high risk of MAFLD and was characterized by decreased DNA methylation (methylation profile 1, driven by lower methylation at *BTF3L*, *HLA-DQB2*, *HSPD1*, and *EPM2AIP1*), lower miRNA levels (miRNA profile 1, driven by lower levels of miR-101–3p, miR-19b-3p, miR-21–5p, and miR-142–3p) and increased gene transcription levels (transcript profile 1, driven by higher *BEND4* and *SLC9A3*). In contrast, group 6 had slightly lower mercury exposure, but considerably higher childhood CK-18 compared to Group 8. The only difference in omics profiles between these groups was that group 6 had higher levels of several miRNAs (miRNA profile 0). This suggests that alterations in miRNA expression could play a role in differentiating between moderate or severe risk of MAFLD in individuals with high prenatal mercury exposure.

Quasi-mediation analysis with late integration (Fig. 1I) identified two omics profiles for the methylome, two omic profiles for the transcriptome, and two omic profiles for miRNA that successively linked mercury exposure with childhood CK-18 based on an a-priori-defined biological hypothesis (Table S3). Here, we hypothesized prenatal mercury exposure would alter DNA methylation, successively impacting miRNA levels, gene transcription, and the outcome (Figure S3). First, each SD increase in prenatal mercury exposure was associated with 1.24 times higher odds of inclusion in a latent cluster characterized by differential DNA methylation at six genes (95% CI: 0.70–2.10; p = 0.448). The high-risk methylation cluster was associated with 1.64 times higher odds of inclusion in a high-risk miRNA specific cluster, which was characterized by differential levels of 13 miRNAs (95% CI: 0.51–5.64; p = 0.406). The high-risk miRNA cluster was associated with 2.75 times higher odds of inclusion in a transcriptome latent cluster, characterized by differential levels of four transcripts (95% CI: 0.75–12.94; p = 0.146). Finally, the high-risk transcription cluster, which incorporates information from each previous omic layer, was associated with a 4.95 SD higher CK-18 (95% CI: 4.55–5.36; p < 0.00001). This approach allows for omic layers to influence each other sequentially, thus reflecting either previously known biological relations or temporal measurements of omic layers.

### 3.5. Commonalities across methods

Although the various approaches for mediation with multi-omics each provide unique insight into the mercury-MAFLD association, there were also several similarities. All approaches consistently identified that blood DNA methylation and altered gene transcription were the primary omics layers linking prenatal mercury and childhood MAFLD. For high dimensional mediation analysis, each multi-omic integration approach identified DNA methylation sites and gene transcripts linking prenatal mercury with childhood MAFLD. The strongest association was observed with a hypermethylation at a CpG site associated with the *EPM2AIP1/MLH1* genes, which was identified across early, intermediate, and late integration. Mediation with latent factors found that factors driven by alterations in DNA methylation and gene transcription had the strongest mediation effects across all multi-omic integration approaches. In contrast to other methods, mediation with latent factors using early and intermediate integration also identified factors driven by differential miRNA and metabolite levels, but the factors characterized by these features mediated less of the total effect of mercury on childhood MAFLD compared to those driven by DNA methylation and gene transcription. Finally, for quasi-mediation, intermediate integration demonstrated that mercury-induced alterations in DNA methylation (including hypermethylation at *EPM2AIP1*) had the largest impact on childhood MAFLD compared to mercury-induced alterations in gene transcription or miRNA. The similarities across methods demonstrate that although the interpretation of results across approaches differs, the strongest effects, such as hypermethylation at *EPM2AIP1*, can be identified across all analytical methods.

## 4. Discussion

To our knowledge, no conceptual framework exists for combining methods for high dimensional mediation with methods for integrating multi-omic layers. By applying our novel conceptual framework of multi-omic data integration and mediation analysis to the HELIX cohort, we have illustrated how molecular intermediaries, including DNA methylation, gene expression, miRNA expression, proteomics, and metabolomics, link environmental exposures and risk of disease. Further, we show how to use different approaches for mediation with multiple omic layers to address key aspects of precision health, including environmental disease biomarker discovery, improving mechanistic insight on environment-associated diseases, and identification of high-risk individuals based on integrated environmental and biological risk profiles.

The overarching goal of this manuscript was to demonstrate that by using a variety of existing methods in a structured framework (as described in Fig. 1), we could identify biologically significant insights related to precision environmental health. Each of the statistical methods that was employed was selected because previous simulation studies have demonstrated the analytical robustness of the method. These existing methods were applied to multiomic data which was combined using early, intermediate, or late integration, each of which has different biological assumptions about how the multiple omics datasets are related to each other and statistical assumptions about the dimensionality, correlation structures, variance, and measurement error between different omics layers. While there were many consistent findings across approaches, there were also several unique findings for each method.

In the first component of our framework, we used high dimensional multiomic mediation to identify exposure-disease related biomarkers. This approach relied on HIMA, xtune, and the distribution of the product method; the analytical robustness of these approaches has been previously established (Zhang et al., 2016; Zeng et al., 2021; Tofighi and MacKinnon, 2011). These analyses use penalization to reduce the potential for overfitting the data, which increases the potential to identify surrogate biomarkers that inform on exposure-disease associations (Ma and Huang, 2008). However, this penalization has the potential to remove causal biological features that may be less strongly associated with exposure and outcome, and thus, it is important to validate these results experimentally. For this approach, the most consistent finding was related to hypermethylation at a CpG site associated with *EPM2AIP1*. This gene encodes for Epm2aip1, a protein related to glycogen synthase. Experimental studies have shown that *EPM2AIP1* gene expression decreases following methyl mercury exposure in pluripotent stem cells (Waldmann et al., 2017), and animal studies have demonstrated that lower Epm2aip1 increases hepatic lipid accumulation (Turnbull et al., 2013). Our results, in conjunction with previous experimental data, suggests that DNA methylation at *EPM2AIP1* may be a biomarker of prenatal mercury associated MAFLD.

In the second component of our framework, we used mediation with latent factors to identify biological pathways linking prenatal mercury with MAFLD. This approach relied on PCA, JIVE and HIMA, all existing methods (Lock et al., 2013; Zhang et al., 2016; Pearson, 1901). Across all three multiomic integration methods, pathways related to glucose regulation and insulin signaling were consistently identified. These were driven by alterations in a broad number of features across omics layers. These findings suggest that insulin signaling may be an important biological mechanism linking prenatal mercury with risk of MAFLD. Therapeutic interventions for insulin resistance in children have previously been shown to improve MAFLD severity in children (Turnbull et al., 2013); our findings may present a new avenue for preventing mercury induced MAFLD in children.

In the third component of our framework, we used quasi-mediation to identify multiomic profiles that can be used to identify youth at high risk of mercury induced MAFLD. This approach relied on LUCID, which has been previously validated (Peng et al., 2020). The key features in this analysis were similar to those identified in other methods, but this approach provided additional evidence that the combination of molecular features across omics layers, as opposed to individual biomarkers, can be used to identify high risk adolescents. These risk profiles could potentially be used to identify children at highest risk of mercury related MAFLD, which in conjunction with novel therapeutic approaches identified from mediation with latent factors could be used to prevent the development mercury induced MAFLD.

One of the most consistent observations across all approaches was that alterations in DNA methylation were identified as key factors in mediating the association between prenatal mercury and childhood liver enzymes. This is consistent with previous reports suggesting that inutero mercury exposure can alter proteins responsible for regulating DNA methylation leading to long term adverse health effects (Go et al., 2021). Organomercury compounds including methylmercury, the predominate chemical species of mercury exposure (Mahaffey, 2005), tend to be electrophilic and have a high affinity for thiol containing molecules and proteins, including cysteine, glutathione, and methionine (LaVoie et al., 2015). As such, these compounds can interfere with the function of enzymes containing these amino acids, including DNA methyl-transferases (DNMT) which are responsible for maintaining DNA methylation patterns (Go et al., 2021). In-vitro and in-vivo studies have shown that during fetal development, mercury exposure alters DNMT levels, resulting in alterations in DNA methylation (Go et al., 2021; Baccarelli and Bollati, 2009). Changes in DNA methylation at important genes related to liver function could have an array of adverse health impacts, as described above in relation to the *EPM2AIP1* gene. Our results build on this prior experimental evidence and suggest that mercury induced changes in DNA methylation are also linked to a variety of other changes in molecular profiles that may ultimately impact liver development.

An important aspect of our framework is that it is easily adaptable to various uses and can incorporate a variety of different high-dimensional mediation and/or multi-omic integration methods. For example, we demonstrated mediation with latent factors using the R package JIVE to identify joint and individual omics factors (Lock et al., 2013). However, this could easily be altered to use other existing joint dimensionality reduction methods, such as those described by Cantini et al. (Cantini et al., 2021).

A potential concern when performing a multiomic mediation analysis with a single environmental factor is that other environmental pollutants may have similar causal molecular pathways, and thus, the molecular signatures identified in this type of analysis may not be specific to the single environmental factor included in analysis. One limitation of our study is that it is possible that co-exposure to other environmental pollutants could have similar effects on important biological pathways. For example, it is well known that exposure to several different classes of environmental pollutants, including other metals, air pollution, and persistent organic pollutants, can impact DNA methylation (Baccarelli and Bollati, 2009). While a detailed analysis of the impact of other environmental pollutants is beyond the scope of the current study, when applying our framework, it is important for researchers to carefully consider how these competing environmental pollutants may impact results. With the increasing availability of high dimensional “exposomics” datasets which can inform on a broad range of different environmental factors, it is often possible to perform additional analyses to alleviate some of these concerns. One option is to model groups of environmental pollutants as a mixture to understand how combinations of environmental factors impact molecular signatures and disease risk (described below). Alternatively, when understanding a single environmental exposure is of interest, there are several possible approaches. For example, if the dimensionality of other exposures is relatively low, it is possible to simply include these other exposures as covariates in the mediator-outcome models. When the dimensionality of other exposures is higher, more complex approaches may be needed. For example, to identify biomarkers of exposure associated disease that are specific to an individual exposure, one option is to perform an additional pre-filtration step to exclude omics features associated with other environmental factors. Another option is to validate biological pathways by performing carefully designed experimental models. Finally, validating associations in additional cohorts with different patterns of exposures can help to provide additional evidence for a link between exposures, omics, and outcomes, regardless of the analytic approach.

Our framework can easily be modified to incorporate multiple exposures. One option for expanding our framework to multiple exposures is to perform pairwise mediation analyses for all combinations of exposures and mediators. While this provides exposure- and omic-specific estimates, it also increases the dimensionality of the results, which may make interpretation more difficult. Another option for incorporating multiple exposures within our framework is to perform dimensionality reduction on the exposures. A simple example is to perform principal components analysis on the exposures and take the top principle component as the exposure, but more complex methods also exist, such as constructing environmental risk scores based on prior knowledge or statistical modeling techniques (Aung et al., 2020). The final option for integrating multiple exposures in our framework is specific to the quasi-mediation approach. Quasi-mediation with LUCID can easily be expanded to incorporate multiple exposures because each additional exposure simply requires estimating one additional parameter for each latent cluster variable. When used in an unsupervised model, this approach can also easily be extended to include multiple outcomes by simply estimating the association between the latent cluster and the multiple outcomes.

Our framework can also be expanded beyond environmental exposures. We illustrated our novel framework using prenatal mercury exposure as the environmental factor, a well-known hepatotoxicant chemical. However, our framework can be applied to any risk factor for disease, providing that the risk factor precedes characterization of the omic layer. For example, our framework would work well to examine how genetic risk scores impact multi-omic profiles to affect disease risk, increasing the applicability of our framework beyond environmental epidemiology.

Because our framework is based on a mediation analysis, it is important to understand the potential requirements and additional steps needed to draw causal inferences from the results. In this manuscript, we primarily demonstrated our mediation approaches using HIMA as the final step for selecting significant features or factors mediating the exposure outcome associations. However, when drawing causal inference on the results of mediation analyses is of interest, practitioners may also perform an additional formal causal mediation analysis with the key selected features/factors using either traditional single mediator causal mediation approaches or newer structural equation modeling or Bayesian causal mediation methods which can account for multiple mediators (VanderWeele and Vansteelandt, 2014; VanderWeele, 2016; Albert et al., 2016; Gunzler et al., 2013; Cai et al., 2022; Jérolon et al., 2021). These models estimate direct and indirect effects and help to formalize the assumptions needed for causal interpretations. A general overview of these approaches and their assumptions have been provided previously, but briefly, several assumptions for causal inference of mediation results must be met (VanderWeele, 2016; Pearl, 2010). This includes the assumption that the model is correctly specified (for example, that potential non-linear associations or interactions are correctly modeled), and that there is no unmeasured confounding. Four assumptions related to confounding must be met: 1) no uncontrolled confounding of the exposure-outcome association; 2) no uncontrolled confounding of the mediator-outcome association; 3) no uncontrolled confounding of the exposure-mediator association; and 4) no mediator-outcome confounder that is itself impacted by the exposure (VanderWeele, 2016). In our study, we performed a series of sensitivity analyses related to the association between the exposure and the outcome, which together suggested that the positive association between prenatal mercury and childhood CK-18 is robust to the impact of additional unmeasured confounders and suggests that criteria number 1 holds. However, in general, criteria number 2, 3, and 4 depend to some degree on the omic features that are represented in the mediation analysis. For example, since some epigenetic changes remain relatively constant after birth, it may be more reasonable to assume that the there are no unmeasured confounders of the association between epigenetic changes (measured in childhood) and liver injury (also measured in childhood) that are also caused by prenatal mercury exposure. This assumption may be more difficult to guarantee for other omics layers; for example, the association between childhood metabolite levels and liver injury could potentially be confounded by altered developmental trajectories caused by prenatal mercury exposure. Because of the strict assumptions needed to draw causal inference on the results of our analysis, we view the biological pathways identified from multiomic mediation analyses as primarily hypothesis generating. For the case study presented here, the mediators and the outcome were assessed at the same timepoint, increasing the chances that reverse causality or unmeasured confounders impact this relationship; thus, our primary results should be validated in other cohorts or using experimental studies. However, our results still serve the function of illustrating the utility of different components of our novel analytic framework.

Although strict assumptions must be met to draw causal interpretations from the results of multiomic mediation analyses, using mediation models to identify predictive biomarkers or high-risk groups of children requires less strict assumptions. For example, building upon the example provided above, if prenatal mercury alters developmental trajectories in early life, these developmental trajectories could confound the mediator-outcome association (assumption criteria #4), because it could cause both altered omics profiles and childhood liver injury. However, if the goal is to predict children at high risk of liver disease (by either identifying predictive biomarkers as described in component one of our framework or by identifying groups of high risk children as described in component three of our framework), demonstration of an exposure-omics-outcome association may be sufficient. However, developing robust prediction models requires additional steps to reduce overfitting and ensure transferability between populations, and additional validation data sets, as described previously (Hastie et al., 2009).

Another assumption of our mediation framework is that the distribution of disease in the study is representative of the distribution of disease in the population (i.e., a prospective likelihood). This condition is generally met in prospective studies, but in studies that use retrospective sampling, such as nested case-control designs, this assumption is not met. For these sampling designs, additional approaches are necessary to estimate the direct and indirect effects, such as using inverse probability weighting or g-computation (Vansteelandt, 2009). As with all mediation analyses, it is important to ensure that the key assumptions are met to draw many of the biological interpretations described herein.

While the underlying biological complexity linking multiple omic datasets is more complex than any statistical representation, our overall framework allows the investigator to make decisions balancing analytic simplicity and complexity while remaining connected to the original exposure to outcome hypothesis that frequently guide a study. Although high-dimensional mediation approaches can have numerous parameters, they are often easier to interpret the final selected features. However, high-dimensional mediation also has drawbacks, including low tolerance to sparsity of direct effects, and the dependence of indirect effects on the existence of total effects. Alternative and more complex methods can overcome some of these methods while modeling joint correlation structures, but they often sacrifice interpretability. These tradeoffs extend to approaches not presented within this framework. For example, alternative approaches, such as neural networks and deep learning, could also be applied to integrate environmental exposures and multiple omic layers to predict disease (Reel et al., 2021). However, the results from these models tend to focus primarily on overall prediction and generally do not allow for a straightforward interpretation of the biology underlying exposure-outcome associations. Our quasi-mediation approach also overcomes some limitations of high-dimensional mediation analysis without sacrificing biological interpretability. It can also be used for disease prediction with comparatively good performance with far fewer parameters and computational time than common deep learning models. The different approaches in our framework provide a balance of disease prediction and biological interpretation.

Despite the strengths of our study, there are some limitations worth noting. One limitation is that total mercury was assessed in cord blood and using different methods in INMA versus the other three cohorts included in the study which assessed mercury from whole blood samples during pregnancy. However, despite this difference, there was no statistically significant heterogeneity in associations between prenatal mercury and childhood CK-18 levels across cohorts, suggesting that this difference did not impact the primary associations between the exposure and the outcome.

In summary, we have presented a novel precision health framework to examine the impact of environmental factors on disease risk by integrating data on multiple omics layers simultaneously. The application of this framework identified novel molecular insights into the relationship between prenatal mercury exposure and risk of MAFLD in childhood. It also identified groups of children at highest risk of mercury-induced MAFLD based on integrated environmental and biological factors. Collectively, different aspects of our framework facilitated the discovery of biological insights and identification of molecular profiles of mercury-induced MAFLD, paving the way for innovative therapeutic strategies. Importantly, it is important to note that our framework aims to complement, rather than replace, existing public health measures that focus on reducing overall exposure to environmental pollutants. We believe that this dual approach not only maximizes public health benefits but also ensures that interventions are more equitable. By tailoring interventions to those most at risk, there is increased potential for resources to be used more efficiently, ultimately reducing the burden of environmental pollutants in populations that bear an unequal brunt of their adverse health effects. This could involve targeted health education, specific preventive measures, or customized medical treatments based on the unique molecular profiles identified through this type of research. This framework can serve as a resource for researchers in a variety of fields by demonstrating how different approaches for mediation with multiple omic layers can be used to address different aspects of precision health to realize this goal. The code and reproducible examples for this analytic framework are available in the Supplemental Methods, at https://goodrich-lab.github.io/multiomics_book/, and at https://doi.org/10.5281/zenodo.10067994.

## Supplementary Material

1

2

## Figures and Tables

**Table 1:** Participant characteristics of the 420 mother-child pairs included in the analysis from the four European cohorts.

Characteristic	Value

**Sample size**	420
**Child Age, Mean (SD)**	7.19 (1.11)
**Child Sex, n (%)**	
Female	192 (45.7%)
Male	228 (54.3%)
**HELIX cohort, n (%)**	
BIB	68 (16.2%)
KANC	112 (26.7%)
RHEA	118 (28.1%)
INMA	122 (29.0%)
**Maternal Smoking, n (%)**	
**No**	349 (83.1%)
**Yes**	71 (16.9%)
**Maternal Fish Intake, n (%)**	
<2 times per week	233 (55.5%)
2–4 times per week	88 (21.0%)
>4 times per week	99 (23.5%)
**Mercury (ng/mL), GM (95% CI)**	3.00 (2.84, 3.18)

## Data Availability

Data will be made available on request.
