# Supplementary material for: Integrating Multi-Omics with environmental data for precision health: A novel analytic framework and case study on prenatal mercury induced childhood fatty liver disease
Source: Environ Int. Author manuscript; Available in PMC 2024 Dec 5. (PMC11620538; doi:10.1016/j.envint.2024.108930)

Supplemental Figures

**Table of Contents**

[**Figure S1.** Individual cohort effects for the association between prenatal total mercury and childhood CK-18 levels. 2](#_Toc167776668)

[**Figure S2.** Quasi-mediation analysis with early integration 3](#_Toc167776669)

[**Figure S3.** Quasi-mediation analysis with late integration 4](#_Toc167776670)

**Figure S1.** Individual cohort effects for the association between prenatal total mercury and childhood CK-18 levels. Effect estimates represent the change in standard deviations per one standard deviation increase in prenatal total mercury. Total effect was calculated using a fixed effect inverse variance weighted method. TE: total effect; SE: standard error; IV: inverse variance; CI: confidence interval; BIB: Born in Bradford study; KANC: the Kaunus cohort; RHEA: RHEA Mother Child Cohort; INMA: INfancia y Medio Ambiente cohort.

**
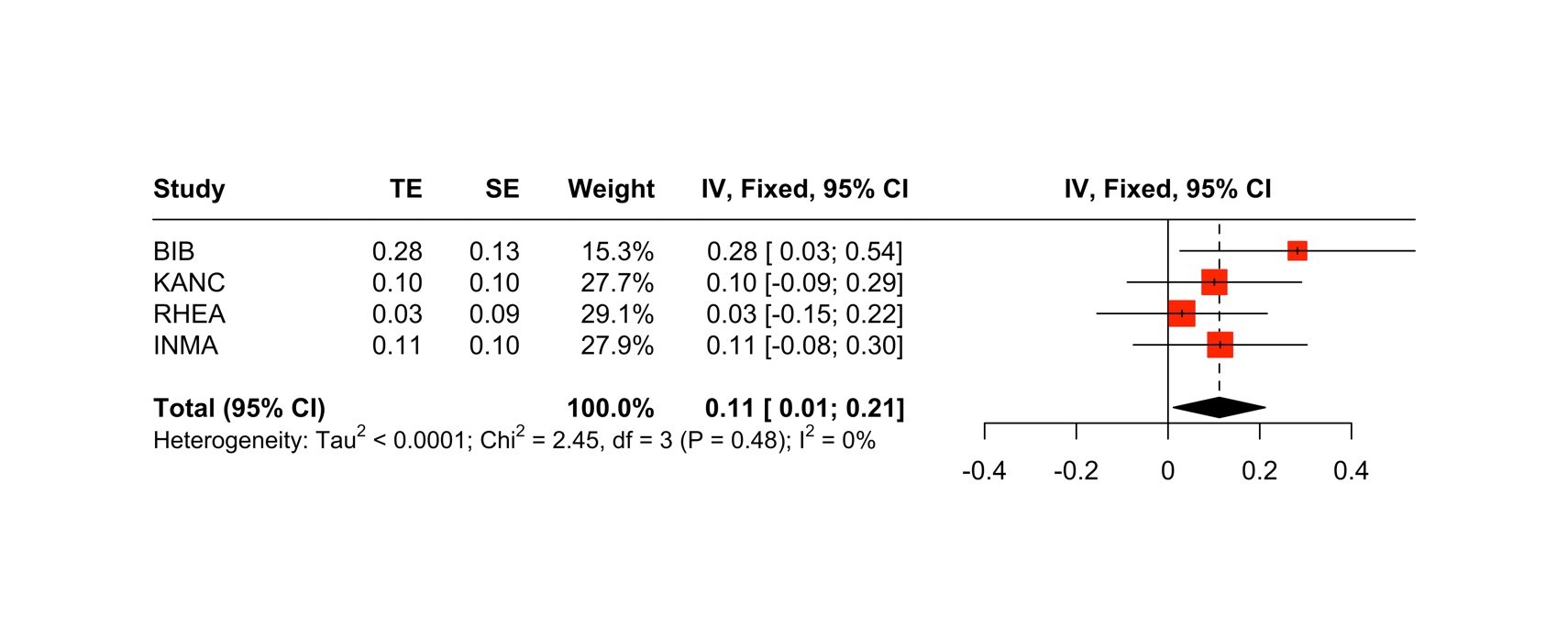
**

**Figure S2.** Quasi-mediation analysis with early integration identifies two groups of children, characterized by joint omics profiles, at risk of MAFLD based on prenatal mercury exposure and distinct omics profiles (Fig 1, Column 3). The figure shows the associations of in utero mercury exposure (on left) with different omics profiles (in the middle). The red line connecting mercury exposure with the joint omics profile 1 indicates a positive association between in utero mercury exposure, with the width of the lines being proportional to the magnitude of the association. The red line connecting the omics profiles with the outcome indicates that these omics profiles are associated with higher risk of liver injury in childhood. The dark green, dark purple, and dark gold lines indicate positive associations between the omic profile and the omic feature, while the light green, light purple, and light gold lines indicate negative associations.


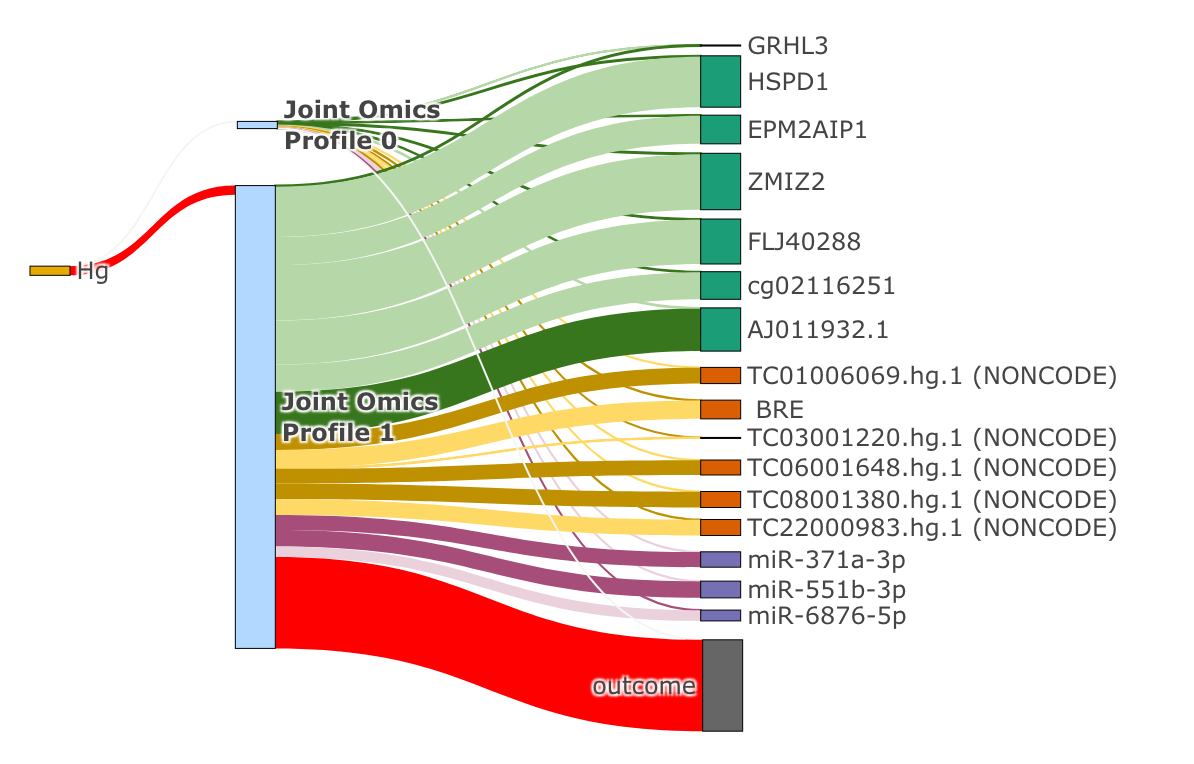


**Figure S3.** Quasi-mediation analysis with late integration illustrates the results of a multiomic analysis based on a-priori biological knowledge. Here, the biological hypothesis is that maternal mercury impacts DNA methylation. DNA methylation in turn impacts miRNA levels and then gene expression, which in turn impacts risk of MAFLD. The figure shows the associations of in utero mercury exposure (on left) with each successive omic layer. The red line connecting mercury exposure with methylation profile 1 indicates a positive association between in utero mercury exposure, with the width of the lines being proportional to the magnitude of the association. The red line connecting the omics profiles with the outcome indicates that these omics profiles are associated with higher risk of liver injury in childhood. The dark green, dark purple, and dark gold lines linking the indicate positive associations between the omic profile and the omic feature or the successive omic profile, while the light green, light purple, and light gold lines indicate negative associations.


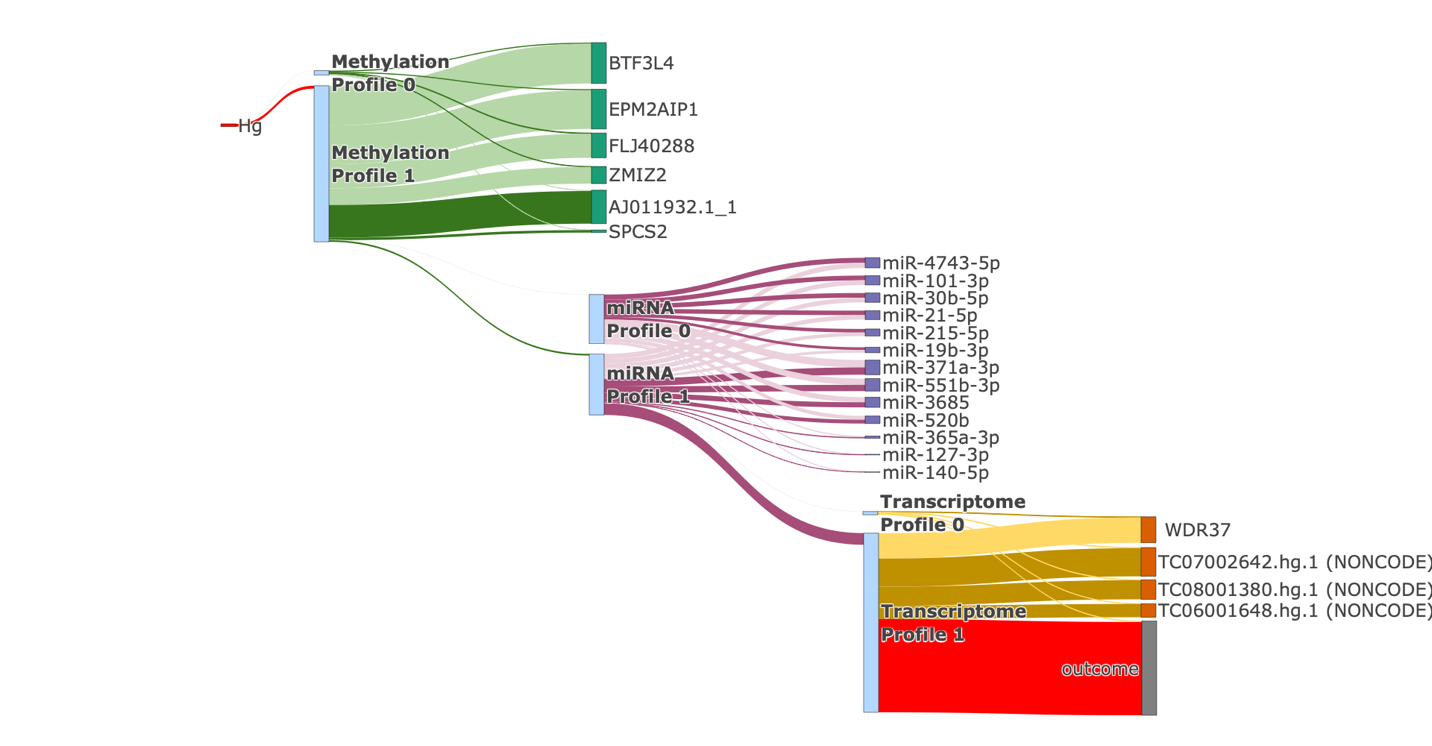

Supplement: 1 [file NIHMS2036061-supplement-1.docx]
