# Supplementary material for: Integrating Multi-Omics with environmental data for precision health: A novel analytic framework and case study on prenatal mercury induced childhood fatty liver disease
Source: Environ Int. Author manuscript; Available in PMC 2024 Dec 5. (PMC11620538; doi:10.1016/j.envint.2024.108930)
Supplement: 2 [file NIHMS2036061-supplement-2.docx]

Supplemental Tables

**Table of Contents**

[Table S1. All significant individual multiomic features identified using high dimensional mediation analysis with multiple omic layers. 2](#_Toc151023460)

[Table S2. Associations of individual features with the individual or joint components identified as significant for mediation analysis with latent factors. Values represent the Pearson correlation coefficient (r) between the individual feature and the individual or joint component. 4](#_Toc151023461)

[Table S3. Mean values (in units of standard deviation) of each individual feature with each of the omic profiles identified using Integrated/Quasi mediation. 9](#_Toc151023462)

# Table S1. All significant individual multiomic features identified using high dimensional mediation analysis with multiple omic layers. Alpha represents the coefficient estimates of the exposure to the mediator, Beta indicates the coefficient estimates of the mediators to the outcome, and TE (%) represents the percent total effect mediated calculated as alpha*beta/gamma. "-" indicates that the effect was estimated to be zero.

|  |  |  | **Early Integration** | | |  | **Intermediate Integration** | | |  | **Late Integration** | | | |
| --- | --- | --- | --- | --- | --- | --- | --- | --- | --- | --- | --- | --- | --- | --- |
| **Omics Layer** | **Feature Name** | **Associated Gene** | **Alpha** | **Beta** | **% TE** |  | **Alpha** | **Beta** | **% TE** |  | **Alpha** | **Beta** | **% TE** |  |
| *DNA Methylation* | *CpG Site* | *Associated Gene* |  |  |  |  |  |  |  |  |  |  |  |  |
|  | cg07948599 | *AL358472.7; CREB3L4; SLC39A1* | -0.152 | -0.082 | 5.6508 |  | - | - | - |  | -0.152 | -0.058 | 2.3638 |  |
|  | cg26853855 | *CSRNP3* | -0.151 | -0.078 | 5.3297 |  | -0.151 | -0.065 | 7.7554 |  | -0.151 | -0.078 | 3.1533 |  |
|  | cg13846866 | *EPM2AIP1;MLH1* | 0.123 | 0.1752 | 9.738 |  | 0.1237 | 0.1435 | 13.942 |  | 0.123 | 0.1919 | 6.2988 |  |
|  | cg08707475 | *AC025171.1;ZNF131* | -0.187 | -0.127 | 10.68 |  | - | - | - |  | -0.187 | -0.09 | 4.4915 |  |
|  | cg04090745 | *HLA-DQB2* | -0.136 | -0.088 | 5.3961 |  | -0.143 | -0.087 | 9.798 |  | -0.136 | -0.138 | 4.9825 |  |
|  | cg02116251 | *-* | -0.119 | -0.19 | 10.249 |  | -0.12 | -0.132 | 12.494 |  | -0.119 | -0.115 | 3.6648 |  |
|  | cg14207210 | *PPM1E* | -0.14 | -0.137 | 8.6794 |  | -0.143 | -0.092 | 10.317 |  | -0.14 | -0.166 | 6.205 |  |
|  | cg05898092 | *DUS1L* | -0.14 | -0.102 | 6.417 |  | -0.139 | -0.086 | 9.3738 |  | -0.14 | -0.076 | 2.8205 |  |
|  | cg07385577 | *AJ011932.1* | 0.1191 | 0.1387 | 7.464 |  | - | - | - |  | - | - | - |  |
|  | cg05762852 | *HDGF;PRCC* | - | - | - |  | - | - | - |  | -0.149 | -0.076 | 3.0372 |  |
|  | cg21941251 | *TDRD5* | - | - | - |  | - | - | - |  | -0.139 | -0.102 | 3.778 |  |
|  | cg24617363 | *NFYA;OARD1* | - | - | - |  | - | - | - |  | 0.154 | 0.1211 | 4.9773 |  |
|  | cg21972156 | *ZMIZ2* | - | - | - |  | - | - | - |  | -0.124 | -0.167 | 5.5187 |  |
|  | cg26182263 | *SLC39A14* | - | - | - |  | - | - | - |  | 0.1296 | 0.0959 | 3.3166 |  |
|  | cg20504025 | *SPCS2;XRRA1* | - | - | - |  | - | - | - |  | -0.102 | -0.138 | 3.7549 |  |
|  | cg14659082 | *AC006538.2;SLC39A3* | - | - | - |  | - | - | - |  | -0.144 | -0.103 | 3.9538 |  |
|  | cg25627242 | *CILP2* | - | - | - |  | - | - | - |  | 0.149 | 0.1355 | 5.3845 |  |
|  | cg08111863 | *SHANK2* | - | - | - |  | -0.166 | -0.062 | 8.0762 |  | - | - | - |  |
| *Gene Transcripts* | *Transcript Name* | *Associated Gene* |  |  |  |  |  |  |  |  |  |  |  |  |
|  | TC02002300.hg.1 | *RAB6C-AS1* | 0.1099 | 0.1001 | 4.9692 |  | 0.1146 | 0.0849 | 7.6405 |  | 0.1099 | 0.1213 | 3.5567 |  |
|  | TC05001096.hg.1 | *SLC9A3* | -0.113 | -0.07 | 3.5753 |  | -0.116 | -0.064 | 5.7907 |  | -0.113 | -0.134 | 4.0526 |  |
|  | TC06001733.hg.1 |  | 0.1296 | 0.0667 | 3.9069 |  | 0.1366 | 0.0731 | 7.843 |  | 0.1296 | 0.1439 | 4.9758 |  |
|  | TC17001096.hg.1 | *LOC284023* | 0.1251 | 0.1301 | 7.3596 |  | - | - | - |  | - | - | - |  |
|  | TC19000509.hg.1 | *ZNF568* | -0.13 | -0.088 | 5.1763 |  | -0.125 | -0.071 | 6.9695 |  | -0.13 | -0.134 | 4.6343 |  |
|  | TC20001151.hg.1 | *-* | -0.118 | -0.101 | 5.4089 |  | - | - | - |  | - | - | - |  |
|  | TC02004659.hg.1 | *LOC100129029* | - | - | - |  | - | - | - |  | 0.1057 | 0.1413 | 3.9843 |  |
|  | TC02004954.hg.1 | *BRE* | - | - | - |  | - | - | - |  | -0.111 | -0.113 | 3.3419 |  |
|  | TC10000010.hg.1 | *WDR37* | - | - | - |  | - | - | - |  | -0.119 | -0.097 | 3.0827 |  |
|  | TC10000169.hg.1 | *KIAA1217* | - | - | - |  | - | - | - |  | 0.1226 | 0.1428 | 4.6705 |  |
| *miRNA* |  |  | *None Identified* | | |  | *None Identified* | | |  | *None Identified* | | | |
| *Proteins* |  |  | *None Identified* | | |  | *None Identified* | | |  | *None Identified* | | | |
| *Metabolite* |  |  | *None Identified* | | |  | *None Identified* | | |  | *None Identified* | | | |

# Table S2. Associations of individual features with the individual or joint components identified as significant for mediation analysis with latent factors. Values represent the Pearson correlation coefficient (r) between the individual feature and the individual or joint component.

|  |  |  | **Early Integration** | | | |  | **Intermediate Integration** | | | | | | | |  | | **Late Integration** | | | | |
| --- | --- | --- | --- | --- | --- | --- | --- | --- | --- | --- | --- | --- | --- | --- | --- | --- | --- | --- | --- | --- | --- | --- |
| **Omic Layer** | **Feature Name** | **Feature information** | **Joint Comp. 1** | **Joint Comp. 2** | **Joint Comp. 4** | **Joint Comp. 8** |  | **Joint Comp. 1** | **Joint Comp. 2** | **Joint Comp. 3** | **Methylome Comp. 1** | **Methylome Comp. 3** | **Transcriptome Comp. 1** | **Transcriptome Comp. 2** |  | | **Methylome Comp. 1** | | **Methylome Comp. 2** | **Methylome Comp. 3** | **Transcriptome Comp. 1** | **Transcriptome Comp. 2** |
| *DNA Methylation* | *CpG Site* | *Associated Gene* |  |  |  |  |  |  |  |  |  |  |  |  |  | |  | |  |  |  |  |
|  | cg00659559 | *SCRN1* | 0.0809 | -0.1465 | -0.0677 | 2.13E-01 |  | 0.0791 | 0.1258 | -0.1006 | -0.0399 | -3.79E-01 |  |  |  | | 0.0815 | | -0.5771 | -3.20E-01 |  |  |
|  | cg01119512 | *GRHL3;GRHL3-AS1* | 0.1102 | -0.0285 | -7.33E-02 | 2.13E-01 |  | 0.1094 | 0.0172 | -0.1967 | -3.50E-03 | -0.2517 |  |  |  | | 6.18E-02 | | -2.34E-01 | 0.2462 |  |  |
|  | cg02053188 | *VTRNA1-3* | -0.0371 | 0.0128 | 0.1529 | -2.19E-01 |  | -0.024 | -0.0022 | 4.17E-01 | 0.0836 | 0.0093 |  |  |  | | -0.1726 | | 0.3002 | -0.1437 |  |  |
|  | cg02096001 | *EIF1* | -0.091 | -0.0107 | 0.6568 | 1.10E-01 |  | -0.0542 | 0.0089 | 0.0782 | 0.7057 | -8.13E-02 |  |  |  | | -0.7067 | | -0.0631 | 5.67E-02 |  |  |
|  | cg02116251 | - | 0.072 | -0.0664 | -1.06E-01 | 0.1127 |  | 0.0719 | 0.0816 | -0.0433 | -7.61E-02 | -0.341 |  |  |  | | 9.54E-02 | | -1.57E-02 | 0.5341 |  |  |
|  | cg04090745 | *HLA-DQB2* | 0.0476 | -0.0737 | -0.454 | -9.47E-02 |  | 0.0257 | 0.0735 | -1.50E-01 | -0.4675 | 1.65E-01 |  |  |  | | 0.4942 | | -0.038 | -1.42E-01 |  |  |
|  | cg05762852 | *HDGF;PRCC* | 0.0126 | -0.1029 | -0.0452 | 3.44E-01 |  | 0.0045 | 0.1 | -2.53E-01 | 0.0329 | -2.70E-01 |  |  |  | | 0.0184 | | -0.3656 | 2.27E-01 |  |  |
|  | cg05773599 | *WDR90* | 0.1417 | 0.0203 | -0.6536 | -6.48E-02 |  | 0.1112 | -0.0264 | -1.53E-01 | -6.95E-01 | -0.0317 |  |  |  | | 0.7299 | | -0.0306 | 0.0329 |  |  |
|  | cg05794325 | *BTF3L4;TXNDC12* | 0.0833 | -0.0196 | -6.75E-01 | -1.64E-01 |  | 0.0506 | 0.0106 | -0.036 | -7.44E-01 | -0.0058 |  |  |  | | 7.46E-01 | | 1.40E-01 | 0.0267 |  |  |
|  | cg05898092 | *DUS1L* | -0.0261 | 0.0108 | -6.95E-02 | 1.82E-01 |  | -0.0264 | -0.0114 | -1.37E-01 | -0.0143 | -2.99E-01 |  |  |  | | 0.0392 | | -0.1126 | 3.44E-01 |  |  |
|  | cg07385577 | *AJ011932.1* | -1.62E-01 | -0.0472 | 7.35E-01 | 0.1334 |  | -0.126 | 0.0561 | 0.1155 | 7.97E-01 | -0.1075 |  |  |  | | -8.11E-01 | | -9.80E-02 | 0.0368 |  |  |
|  | cg07948599 | *AL358472.7;CREB3L4;SLC39A1* | 0.0827 | -0.0152 | -0.121 | 2.60E-01 |  | 0.0814 | 0.0099 | -0.0753 | -0.0931 | -3.86E-01 |  |  |  | | 0.123 | | -0.2352 | 3.53E-01 |  |  |
|  | cg08111863 | *SHANK2* | 0.01 | -0.0408 | -0.1728 | -2.40E-03 |  | -0.0038 | 0.0255 | -0.1715 | -0.1366 | -6.52E-02 |  |  |  | | 0.1773 | | -0.2253 | -1.36E-01 |  |  |
|  | cg08707475 | *AC025171.1;ZNF131* | 0.0981 | -0.0419 | -0.0429 | 2.02E-01 |  | 0.0919 | 0.0399 | -2.31E-01 | 0.0095 | -0.0017 |  |  |  | | 0.0452 | | -0.2637 | 0.0125 |  |  |
|  | cg13846866 | *EPM2AIP1;MLH1* | 0.0428 | 0.0043 | 6.61E-02 | -0.2372 |  | 0.0448 | 0.0117 | 0.1826 | 6.40E-03 | 0.3263 |  |  |  | | -3.43E-02 | | 1.74E-01 | -0.2922 |  |  |
|  | cg13971502 | *COL6A1* | -0.0926 | -0.0543 | 0.5504 | 3.89E-02 |  | -0.0636 | 0.0547 | 2.17E-01 | 0.5536 | -0.172 |  |  |  | | -0.5823 | | 0.0633 | 0.1762 |  |  |
|  | cg14207210 | *PPM1E* | 0.0115 | -0.1011 | 0.0393 | 2.75E-01 |  | 0.0106 | 0.0817 | -8.69E-02 | 0.0864 | -0.4341 |  |  |  | | -0.0508 | | -0.588 | -3.24E-01 |  |  |
|  | cg14659082 | *AC006538.2;SLC39A3* | 0.0103 | -0.0308 | -0.0687 | 2.85E-01 |  | 0.0157 | 0.0394 | -2.72E-01 | 0.024 | -1.76E-01 |  |  |  | | 0.0258 | | -0.2786 | 2.71E-01 |  |  |
|  | cg16690859 | *FLJ40288;PLXNA4* | 0.1314 | -0.0118 | -0.8162 | -1.93E-01 |  | 0.0881 | 0.0028 | -5.70E-02 | -0.9114 | 1.13E-02 |  |  |  | | 0.9146 | | 0.1292 | -1.20E-03 |  |  |
|  | cg19965941 | *HSPD1;HSPE1-MOB4;MOB4* | 0.1156 | -0.0349 | -6.06E-01 | -0.0102 |  | 0.0884 | 0.0157 | -0.0941 | -6.55E-01 | -0.0908 |  |  |  | | 6.72E-01 | | -1.86E-01 | -2.21E-01 |  |  |
|  | cg20504025 | *SPCS2;XRRA1* | 0.086 | -0.0836 | -0.1122 | 0.2213 |  | 0.0862 | 0.0916 | -0.3528 | -0.012 | 2.30E-01 |  |  |  | | 0.0763 | | -0.3056 | -7.74E-02 |  |  |
|  | cg21941251 | *TDRD5* | 0.0887 | -0.0195 | -0.1008 | 1.74E-01 |  | 0.0925 | 0.0242 | -2.60E-01 | -0.0382 | -0.0583 |  |  |  | | 0.1052 | | -0.2199 | 8.13E-02 |  |  |
|  | cg21972156 | *ZMIZ2* | 0.0834 | -0.0455 | -7.62E-01 | -0.1498 |  | 0.0426 | 0.043 | -0.0612 | -8.12E-01 | -0.1337 |  |  |  | | 8.16E-01 | | 1.03E-01 | 0.1552 |  |  |
|  | cg24617363 | *NFYA;OARD1* | -0.1306 | 0.0285 | -2.72E-02 | -0.2597 |  | -0.1329 | -0.027 | 0.4233 | -9.05E-02 | -2.11E-01 |  |  |  | | -1.04E-02 | | 3.12E-01 | -0.0931 |  |  |
|  | cg25627242 | *CILP2* | -0.0925 | 0.1412 | 0.0348 | -0.2147 |  | -0.0924 | -0.1429 | 0.3402 | -0.0701 | -1.12E-01 |  |  |  | | -0.0071 | | 0.4223 | 1.47E-01 |  |  |
|  | cg25823142 | *AJ011932.1* | -0.0494 | 0.0481 | 6.23E-01 | 0.1002 |  | -0.0153 | -0.0409 | 0.0738 | 6.48E-01 | 0.0097 |  |  |  | | -6.51E-01 | | -5.16E-02 | 0.0085 |  |  |
|  | cg26182263 | *SLC39A14* | -0.0544 | 0.0781 | 1.14E-01 | -0.154 |  | -0.0514 | -0.0694 | 1.12E-01 | 5.10E-02 | 0.3519 |  |  |  | | -0.0786 | | 0.1664 | -0.3214 |  |  |
|  | cg26853855 | *CSRNP3* | 0.019 | 0.0439 | -1.91E-01 | 0.1926 |  | 0.0081 | -0.0472 | -1.76E-01 | -1.28E-01 | -2.95E-01 |  |  |  | | 1.54E-01 | | -3.91E-02 | 4.17E-01 |  |  |
| *Gene Transcription* | *Transcript Name* | *Associated Gene* |  |  |  |  |  |  |  |  |  |  |  |  |  | |  | |  |  |  |  |
|  | TC01006069.hg.1 | TC01006069.hg.1 (NONCODE) | -0.0255 | -0.0326 | 0.0893 | -2.03E-01 |  | -0.0171 | 0.0343 | 0.0633 |  |  | -1.14E-01 | -3.77E-01 |  | |  | |  |  | 0.2433 | -2.52E-01 |
|  | TC02000627.hg.1 | *SLC9A4* | 0.087 | -0.0504 | -0.0407 | 0.1506 |  | 0.0682 | 0.0344 | 0.0358 |  |  | -0.0274 | 8.29E-02 |  | |  | |  |  | -0.1237 | 0.1768 |
|  | TC02002300.hg.1 | *RAB6C-AS1* | -0.0531 | -0.0018 | -0.0098 | -1.77E-01 |  | -0.0486 | 0.0162 | 0.0401 |  |  | -9.66E-02 | -3.48E-01 |  | |  | |  |  | 0.1913 | -1.85E-01 |
|  | TC02004659.hg.1 | *LOC100129029* | 0.0462 | 0.1101 | 0.0872 | -0.1591 |  | 0.0534 | -0.114 | 1.81E-01 |  |  | 0.1187 | -5.15E-02 |  | |  | |  |  | 0.0774 | -0.2812 |
|  | TC02004954.hg.1 | *BRE* | 0.0258 | 0.0106 | -0.0963 | 0.1202 |  | 0.0307 | -0.0076 | -2.81E-01 |  |  | 8.33E-02 | 0.0071 |  | |  | |  |  | -0.1829 | 0.1236 |
|  | TC03001220.hg.1 | TC03001220.hg.1 (NONCODE) | -0.0254 | 0.0322 | 0.1452 | -2.35E-01 |  | -0.0157 | -0.0125 | 1.61E-01 |  |  | -5.58E-02 | -9.75E-02 |  | |  | |  |  | 0.2561 | -0.2264 |
|  | TC04002114.hg.1 | TC04002114.hg.1 (NONCODE) | -0.1026 | 0.0437 | 2.07E-01 | -1.60E-01 |  | -0.0849 | -0.0402 | 2.90E-01 |  |  | -4.33E-02 | 0.2766 |  | |  | |  |  | 0.217 | -8.33E-02 |
|  | TC04002369.hg.1 | TC04002369.hg.1 (NONCODE) | -0.0781 | -0.0126 | 0.0791 | -2.39E-01 |  | -0.0774 | 0.0161 | 1.72E-01 |  |  | -5.31E-02 | -2.46E-01 |  | |  | |  |  | 0.2112 | -2.30E-01 |
|  | TC04002528.hg.1 | *BEND4* | -0.0437 | -0.0346 | 0.103 | -0.1264 |  | -0.0404 | 0.0436 | 2.47E-01 |  |  | -9.47E-02 | -1.06E-01 |  | |  | |  |  | 0.2089 | -0.1654 |
|  | TC05001096.hg.1 | *SLC9A3* | 0.0093 | 0.0292 | -0.1291 | 0.1309 |  | 0.0033 | -0.029 | -0.1267 |  |  | -0.0089 | 1.74E-01 |  | |  | |  |  | -0.1121 | 2.05E-01 |
|  | TC05001376.hg.1 | TC05001376.hg.1 (NONCODE) | -0.0922 | 0.0778 | 0.0099 | -1.93E-01 |  | -0.0887 | -0.0664 | 2.78E-01 |  |  | -6.98E-02 | 0.2632 |  | |  | |  |  | 0.1787 | -1.58E-02 |
|  | TC06001648.hg.1 | TC06001648.hg.1 (NONCODE) | -0.0239 | 0.064 | 0.0407 | -2.36E-01 |  | -0.0176 | -0.057 | 0.0921 |  |  | -1.97E-01 | -3.92E-01 |  | |  | |  |  | 3.06E-01 | -2.04E-01 |
|  | TC06001733.hg.1 | TC06001733.hg.1 (NONCODE) | -0.0663 | -0.0331 | 0.0029 | -0.1513 |  | -0.0677 | 0.0284 | 1.70E-01 |  |  | 0.1918 | 1.25E-01 |  | |  | |  |  | -1.12E-02 | -0.2252 |
|  | TC06001809.hg.1 | *ICK* | -0.0303 | 0.0638 | 0.0274 | -2.29E-01 |  | -0.0342 | -0.0567 | 1.98E-01 |  |  | -0.0339 | -0.058 |  | |  | |  |  | 0.1539 | -0.1358 |
|  | TC07000748.hg.1 | *HYALP1* | -0.0936 | 0.0334 | 1.98E-01 | -2.10E-01 |  | -0.0766 | -0.0272 | 0.083 |  |  | -1.15E-01 | -2.47E-01 |  | |  | |  |  | 0.287 | -2.15E-01 |
|  | TC07002642.hg.1 | TC07002642.hg.1 (NONCODE) | 0.0243 | 0.0605 | 0.0833 | -1.65E-01 |  | 0.0291 | -0.0532 | 0.1174 |  |  | 0.0348 | -4.20E-01 |  | |  | |  |  | 0.1549 | -3.72E-01 |
|  | TC08000864.hg.1 | TC08000864.hg.1 (NONCODE) | -0.1049 | 0.0659 | -0.0021 | -2.65E-01 |  | -0.0959 | -0.0726 | 3.04E-01 |  |  | -8.96E-02 | -1.04E-01 |  | |  | |  |  | 0.2279 | -0.1859 |
|  | TC08001380.hg.1 | TC08001380.hg.1 (NONCODE) | -0.0982 | 0.0946 | 0.0156 | -2.21E-01 |  | -0.0939 | -0.0814 | 0.0747 |  |  | -2.45E-01 | -0.3095 |  | |  | |  |  | 3.19E-01 | -8.79E-02 |
|  | TC09001768.hg.1 | *C9orf173-AS1* | 0.1095 | -0.0713 | -0.1142 | 3.74E-01 |  | 0.089 | 0.0536 | 0.0135 |  |  | 9.14E-01 | -1.87E-01 |  | |  | |  |  | -7.57E-01 | -5.82E-01 |
|  | TC09002871.hg.1 | *C9orf173-AS1* | 0.0722 | -0.0551 | -0.1487 | 3.64E-01 |  | 0.0505 | 0.0391 | 0.0045 |  |  | 9.11E-01 | -2.21E-01 |  | |  | |  |  | -7.44E-01 | -6.05E-01 |
|  | TC10000010.hg.1 | *WDR37* | -0.003 | -0.0711 | -1.99E-01 | 0.1516 |  | -0.0184 | 0.0679 | -1.99E-01 |  |  | 9.86E-02 | 2.94E-01 |  | |  | |  |  | -0.2257 | 1.77E-01 |
|  | TC10000169.hg.1 | *KIAA1217* | -0.0141 | -0.0691 | 0.1476 | -2.32E-01 |  | -0.0137 | 0.0576 | 2.72E-01 |  |  | -1.40E-01 | -0.0189 |  | |  | |  |  | 0.2022 | 0.0068 |
|  | TC10002518.hg.1 | *LINC00614* | 0.0143 | -0.0532 | 1.76E-01 | -0.1255 |  | 0.0152 | 0.0627 | 0.1563 |  |  | -4.70E-02 | -4.00E-01 |  | |  | |  |  | 0.1996 | -3.12E-01 |
|  | TC12001689.hg.1 | *GRIP1* | 0.0965 | -0.0904 | -0.1242 | 0.0741 |  | 0.0891 | 0.0756 | -0.0978 |  |  | 0.0126 | 1.14E-01 |  | |  | |  |  | -0.1599 | 0.1992 |
|  | TC17001096.hg.1 | *LOC284023* | -0.0245 | 0.0133 | 0.0657 | -0.1598 |  | -0.0145 | -0.0137 | 0.1455 |  |  | -1.55E-01 | -0.0913 |  | |  | |  |  | 0.2427 | -0.1199 |
|  | TC19000509.hg.1 | *ZNF568* | 0.0444 | -0.096 | -0.0787 | 0.1143 |  | 0.0518 | 0.1042 | -2.22E-01 |  |  | -0.0654 | -0.0471 |  | |  | |  |  | -0.0819 | 0.1549 |
|  | TC20001151.hg.1 | TC20001151.hg.1 (NONCODE) | 0.055 | -0.0126 | -1.76E-01 | 1.82E-01 |  | 0.0568 | 0.0117 | -2.11E-01 |  |  | 9.42E-02 | 1.94E-02 |  | |  | |  |  | -0.2505 | 0.1999 |
|  | TC22000983.hg.1 | TC22000983.hg.1 (NONCODE) | 0.0207 | -0.0307 | -0.1048 | 2.45E-01 |  | 0.0111 | 0.0345 | -1.94E-01 |  |  | 1.37E-01 | 2.47E-01 |  | |  | |  |  | -0.2569 | 1.86E-01 |
| *miRNA* | *miRNA Name* | *miRNA Name* |  |  |  |  |  |  |  |  |  |  |  |  |  | |  | |  |  |  |  |
|  | miR-101-3p | hsa-miR-101-3p | -0.1401 | -8.61E-01 | 0.0163 | -0.0467 |  | -0.1462 | 8.55E-01 | 0.0404 |  |  |  |  |  | |  | |  |  |  |  |
|  | miR-125a-5p | hsa-miR-125a-5p | -0.0566 | -4.32E-01 | 0.0166 | -0.1133 |  | -0.0498 | 3.92E-01 | 0.1296 |  |  |  |  |  | |  | |  |  |  |  |
|  | miR-125b-1-3p | hsa-miR-125b-1-3p | -0.1373 | -0.1159 | -0.047 | -1.96E-01 |  | -0.1474 | 0.0905 | 1.78E-01 |  |  |  |  |  | |  | |  |  |  |  |
|  | miR-127-3p | hsa-miR-127-3p | 0.043 | -0.1067 | 0.004 | 0.0733 |  | 0.0373 | 0.1007 | -0.0413 |  |  |  |  |  | |  | |  |  |  |  |
|  | miR-140-5p | hsa-miR-140-5p | -0.1534 | -7.62E-01 | 0.0388 | 0.0162 |  | -0.1499 | 7.77E-01 | 0.051 |  |  |  |  |  | |  | |  |  |  |  |
|  | miR-142-3p | hsa-miR-142-3p | -0.1029 | -8.67E-01 | -0.0187 | 0.0059 |  | -0.1112 | 8.96E-01 | 0.0236 |  |  |  |  |  | |  | |  |  |  |  |
|  | miR-144-5p | hsa-miR-144-5p | -0.1023 | -8.04E-01 | -0.0237 | 0.0602 |  | -0.1033 | 8.41E-01 | 0.0101 |  |  |  |  |  | |  | |  |  |  |  |
|  | miR-19a-3p | hsa-miR-19a-3p | -0.0957 | -8.18E-01 | 0.0064 | 0.0397 |  | -0.0969 | 8.44E-01 | -0.0212 |  |  |  |  |  | |  | |  |  |  |  |
|  | miR-19b-3p | hsa-miR-19b-3p | -0.1508 | -9.25E-01 | -0.0413 | -0.001 |  | -0.155 | 9.42E-01 | 0.0147 |  |  |  |  |  | |  | |  |  |  |  |
|  | miR-21-5p | hsa-miR-21-5p | -0.1474 | -8.99E-01 | -0.0449 | -0.023 |  | -0.1524 | 9.08E-01 | 0.0556 |  |  |  |  |  | |  | |  |  |  |  |
|  | miR-2110 | hsa-miR-2110 | -0.0615 | -4.46E-01 | 0.002 | -0.0969 |  | -0.0516 | 3.64E-01 | -0.0912 |  |  |  |  |  | |  | |  |  |  |  |
|  | miR-215-5p | hsa-miR-215-5p | -0.1385 | -8.81E-01 | -0.0349 | -0.0453 |  | -0.1392 | 8.75E-01 | 0.0629 |  |  |  |  |  | |  | |  |  |  |  |
|  | miR-27a-3p | hsa-miR-27a-3p | -0.1091 | -8.57E-01 | -0.0295 | -0.0733 |  | -0.1089 | 8.54E-01 | 0.0645 |  |  |  |  |  | |  | |  |  |  |  |
|  | miR-29c-3p | hsa-miR-29c-3p | -0.1492 | -7.18E-01 | 0.0203 | -0.0756 |  | -0.1498 | 7.27E-01 | 0.0244 |  |  |  |  |  | |  | |  |  |  |  |
|  | miR-30b-5p | hsa-miR-30b-5p | -0.1582 | -8.68E-01 | -0.0462 | -0.03 |  | -1.64E-01 | 8.65E-01 | 0.0424 |  |  |  |  |  | |  | |  |  |  |  |
|  | miR-331-3p | hsa-miR-331-3p | -0.1099 | -7.78E-01 | 0.0682 | -0.0585 |  | -0.1088 | 7.23E-01 | -0.0436 |  |  |  |  |  | |  | |  |  |  |  |
|  | miR-3614-5p | hsa-miR-3614-5p | 0.0442 | -0.0462 | 0.1015 | 1.81E-01 |  | 0.0467 | 0.0408 | -2.60E-01 |  |  |  |  |  | |  | |  |  |  |  |
|  | miR-365a-3p | hsa-miR-365a-3p | -0.061 | -6.12E-01 | -0.0049 | 0.0039 |  | -0.0521 | 6.01E-01 | 0.0536 |  |  |  |  |  | |  | |  |  |  |  |
|  | miR-365b-5p | hsa-miR-365b-5p | 0.0915 | 0.1226 | -0.122 | -0.0827 |  | 0.0944 | -0.1152 | 0.0226 |  |  |  |  |  | |  | |  |  |  |  |
|  | miR-3685 | hsa-miR-3685 | 0.0271 | -0.035 | 0.0863 | -0.026 |  | 0.0244 | 0.0448 | 0.1161 |  |  |  |  |  | |  | |  |  |  |  |
|  | miR-371a-3p | hsa-miR-371a-3p | -0.0926 | 0.0702 | 2.12E-01 | 0.1583 |  | -0.0842 | -0.0567 | 0.0056 |  |  |  |  |  | |  | |  |  |  |  |
|  | miR-4743-5p | hsa-miR-4743-5p | 0.0345 | 0.1151 | -2.05E-01 | -0.0561 |  | 0.0223 | -0.1193 | -0.0475 |  |  |  |  |  | |  | |  |  |  |  |
|  | miR-486-5p | hsa-miR-486-5p | -0.0459 | -4.80E-01 | 0.1474 | -0.0066 |  | -0.0378 | 4.10E-01 | -0.128 |  |  |  |  |  | |  | |  |  |  |  |
|  | miR-520b | hsa-miR-520b | -0.0349 | 1.69E-01 | 0.0218 | 0.1269 |  | -0.0329 | -1.62E-01 | 0.0597 |  |  |  |  |  | |  | |  |  |  |  |
|  | miR-551b-3p | hsa-miR-551b-3p | -0.0713 | 0.0313 | 0.1011 | -0.0189 |  | -0.071 | -0.0296 | 2.66E-01 |  |  |  |  |  | |  | |  |  |  |  |
|  | miR-6073 | hsa-miR-6073 | 0.0952 | -1.97E-01 | -0.0178 | 0.0511 |  | 0.0968 | 0.1534 | -0.109 |  |  |  |  |  | |  | |  |  |  |  |
|  | miR-628-3p | hsa-miR-628-3p | -0.1132 | -0.1435 | -0.0576 | -0.0398 |  | -0.1128 | 0.1211 | 0.0113 |  |  |  |  |  | |  | |  |  |  |  |
|  | miR-6876-5p | hsa-miR-6876-5p | 0.115 | -0.0898 | -0.0276 | 0.105 |  | 0.1263 | 0.0706 | -2.41E-01 |  |  |  |  |  | |  | |  |  |  |  |
| *Proteins* | *Protein Name* |  |  |  |  |  |  |  |  |  |  |  |  |  |  | |  | |  |  |  |  |
|  | Adiponectin |  | -0.0773 | 0.0222 | -0.0613 | 2.05E-01 |  | -0.0698 | -0.0165 | -0.0364 |  |  |  |  |  | |  | |  |  |  |  |
|  | APO.A1 |  | -2.85E-01 | 0.0257 | -0.0001 | 0.1123 |  | -2.64E-01 | -0.032 | -0.0236 |  |  |  |  |  | |  | |  |  |  |  |
|  | APO.B |  | -0.1081 | 0.0544 | 0.0437 | 0.0745 |  | -0.0852 | -0.0455 | 0.0199 |  |  |  |  |  | |  | |  |  |  |  |
|  | APO.E |  | -0.1076 | 0.0105 | -0.0894 | 0.0531 |  | -0.0819 | -0.0035 | 0.0154 |  |  |  |  |  | |  | |  |  |  |  |
|  | BAFF |  | 0.0185 | 0.0351 | -2.47E-01 | 0.0987 |  | 0.0241 | 0.0012 | 0.0304 |  |  |  |  |  | |  | |  |  |  |  |
|  | Cpeptide |  | 0.0852 | 0.0363 | -0.0371 | 0.1161 |  | 0.0849 | -0.0245 | -0.001 |  |  |  |  |  | |  | |  |  |  |  |
|  | CRP |  | -0.0252 | 0.0551 | 0.0272 | -0.1061 |  | -0.0028 | -0.0471 | 0.0723 |  |  |  |  |  | |  | |  |  |  |  |
|  | EGF |  | 0.0598 | 0.0605 | -1.96E-01 | -0.0728 |  | 0.0387 | -0.0356 | 0.1478 |  |  |  |  |  | |  | |  |  |  |  |
|  | GCSF |  | -0.0148 | 0.0705 | -0.0821 | -0.0624 |  | -0.0284 | -0.0637 | 0.0296 |  |  |  |  |  | |  | |  |  |  |  |
|  | HGF |  | -0.0717 | 0.0164 | -2.76E-01 | 0.0867 |  | -0.077 | 0.0126 | 0.0641 |  |  |  |  |  | |  | |  |  |  |  |
|  | IFNalfa |  | 0.0212 | 0.0551 | -0.1306 | 0.0513 |  | 0.0023 | -0.0326 | -0.0611 |  |  |  |  |  | |  | |  |  |  |  |
|  | IL10 |  | -0.0106 | 0.0448 | -0.1127 | -0.0325 |  | -0.0308 | -0.0291 | 0.111 |  |  |  |  |  | |  | |  |  |  |  |
|  | IL13 |  | 0.103 | 0.015 | -0.127 | 0.0288 |  | 0.0815 | 0.0112 | 0.0467 |  |  |  |  |  | |  | |  |  |  |  |
|  | IL15 |  | -0.0017 | 0.0903 | -0.0933 | 0.0148 |  | -0.0231 | -0.0747 | 0.1165 |  |  |  |  |  | |  | |  |  |  |  |
|  | IL17 |  | 0.0373 | 0.0362 | -0.1123 | 0.0199 |  | 0.0158 | -0.0193 | -0.0399 |  |  |  |  |  | |  | |  |  |  |  |
|  | IL1beta |  | -0.0009 | 0.0093 | -2.14E-01 | -0.053 |  | 0.0045 | 0.019 | 0.1005 |  |  |  |  |  | |  | |  |  |  |  |
|  | IL1RA |  | 0.1105 | 0.0578 | -0.1142 | -0.1351 |  | 0.0983 | -0.026 | 0.1033 |  |  |  |  |  | |  | |  |  |  |  |
|  | IL2R |  | 0.1128 | 0.0369 | -0.0954 | 0.0907 |  | 0.0925 | -0.0144 | -0.127 |  |  |  |  |  | |  | |  |  |  |  |
|  | IL6 |  | 0.014 | 0.0029 | -1.98E-01 | -0.0768 |  | 0.0152 | 0.0273 | 0.091 |  |  |  |  |  | |  | |  |  |  |  |
|  | IL8 |  | -0.0961 | 0.1005 | -2.14E-01 | 0.0062 |  | -0.0962 | -0.0707 | 0.1499 |  |  |  |  |  | |  | |  |  |  |  |
|  | INSULIN |  | -0.0124 | 0.0714 | 0.0131 | 0.1527 |  | -0.0198 | -0.0545 | 0.0763 |  |  |  |  |  | |  | |  |  |  |  |
|  | IP10 |  | 0.0765 | 0.0693 | -0.0424 | -0.0677 |  | 0.0766 | -0.0494 | -0.0663 |  |  |  |  |  | |  | |  |  |  |  |
|  | Leptin |  | -0.0931 | -0.0267 | -0.1356 | -0.109 |  | -0.0982 | 0.0443 | 0.0822 |  |  |  |  |  | |  | |  |  |  |  |
|  | MCP1 |  | -0.0126 | -0.0013 | -2.17E-01 | 0.0158 |  | -0.0192 | 0.02 | -0.123 |  |  |  |  |  | |  | |  |  |  |  |
|  | MIG |  | 0.1027 | 0.1028 | -0.063 | -0.0036 |  | 0.0779 | -0.0795 | -0.0021 |  |  |  |  |  | |  | |  |  |  |  |
|  | MIP1beta |  | 0.0317 | -0.0115 | -0.1449 | -0.0277 |  | 0.0097 | 0.0459 | 0.0212 |  |  |  |  |  | |  | |  |  |  |  |
|  | PAI1 |  | 0.1088 | -0.0532 | 0.0199 | 2.22E-01 |  | 0.1129 | 0.0678 | -0.1519 |  |  |  |  |  | |  | |  |  |  |  |
|  | TNFalfa |  | -0.0009 | 0.0737 | -2.32E-01 | 0.0643 |  | -0.0044 | -0.0449 | 0.0562 |  |  |  |  |  | |  | |  |  |  |  |
| *Metabolites* | *Metabolite Name* |  |  |  |  |  |  |  |  |  |  |  |  |  |  | |  | |  |  |  |  |
|  | C12:1 |  | -0.1509 | 0.022 | -0.0503 | -2.93E-01 |  | -0.1177 | -0.0239 | 0.0454 |  |  |  |  |  | |  | |  |  |  |  |
|  | C16:2 |  | -0.0804 | 0.0086 | 0.0284 | -2.48E-01 |  | -0.0519 | -0.0097 | 0.0631 |  |  |  |  |  | |  | |  |  |  |  |
|  | C5 |  | -0.1433 | 0.0268 | 0.0523 | -0.0488 |  | -0.0777 | -0.0133 | 0.0966 |  |  |  |  |  | |  | |  |  |  |  |
|  | H1 |  | -3.22E-01 | 0.0704 | 0.0942 | 0.0636 |  | -3.55E-01 | -0.072 | 0.0256 |  |  |  |  |  | |  | |  |  |  |  |
|  | PC aa C32:0 |  | -7.40E-01 | 0.0737 | -0.0279 | 0.0188 |  | -7.24E-01 | -0.0801 | -0.0901 |  |  |  |  |  | |  | |  |  |  |  |
|  | PC aa C32:3 |  | -6.88E-01 | 0.0419 | -0.0391 | -0.0338 |  | -6.48E-01 | -0.0406 | 0.0169 |  |  |  |  |  | |  | |  |  |  |  |
|  | PC aa C36:0 |  | -8.52E-01 | 0.0817 | -0.0636 | 0.069 |  | -8.56E-01 | -0.0893 | -0.0938 |  |  |  |  |  | |  | |  |  |  |  |
|  | PC aa C36:5 |  | -6.29E-01 | 0.0651 | -0.0609 | 0.0255 |  | -6.00E-01 | -0.0525 | 0.0545 |  |  |  |  |  | |  | |  |  |  |  |
|  | PC aa C36:6 |  | -7.04E-01 | 0.0256 | -0.0827 | -0.0069 |  | -6.79E-01 | -0.0127 | 0.0444 |  |  |  |  |  | |  | |  |  |  |  |
|  | PC aa C38:0 |  | -8.53E-01 | 0.0941 | -0.0383 | 0.0353 |  | -8.69E-01 | -0.0983 | -0.0479 |  |  |  |  |  | |  | |  |  |  |  |
|  | PC aa C38:1 |  | -6.01E-01 | 0.0709 | -0.0247 | 0.1422 |  | -5.96E-01 | -0.0555 | -0.0397 |  |  |  |  |  | |  | |  |  |  |  |
|  | PC aa C38:6 |  | -7.61E-01 | 0.0952 | -0.029 | 0.051 |  | -7.63E-01 | -0.0908 | -0.0256 |  |  |  |  |  | |  | |  |  |  |  |
|  | PC aa C40:1 |  | -7.58E-01 | 0.0805 | -0.0493 | 0.0892 |  | -7.72E-01 | -0.0799 | -0.0383 |  |  |  |  |  | |  | |  |  |  |  |
|  | PC aa C40:6 |  | -7.25E-01 | 0.0721 | -0.0423 | 0.0526 |  | -7.28E-01 | -0.062 | -0.0003 |  |  |  |  |  | |  | |  |  |  |  |
|  | PC aa C42:0 |  | -7.13E-01 | 0.1544 | -0.049 | 0.0806 |  | -7.42E-01 | -0.1595 | -0.063 |  |  |  |  |  | |  | |  |  |  |  |
|  | PC ae C32:1 |  | -7.56E-01 | 0.0331 | -0.0822 | -0.0102 |  | -7.54E-01 | -0.0452 | -0.0983 |  |  |  |  |  | |  | |  |  |  |  |
|  | PC ae C32:2 |  | -7.97E-01 | 0.0586 | -0.0977 | -0.0168 |  | -7.87E-01 | -0.0615 | -0.0407 |  |  |  |  |  | |  | |  |  |  |  |
|  | PC ae C34:1 |  | -7.64E-01 | 0.0428 | 0.0097 | 0.0186 |  | -7.34E-01 | -0.0478 | -0.0799 |  |  |  |  |  | |  | |  |  |  |  |
|  | PC ae C36:3 |  | -6.95E-01 | 0.0028 | -0.0254 | 0.0004 |  | -6.85E-01 | -0.0059 | -0.0555 |  |  |  |  |  | |  | |  |  |  |  |
|  | PC ae C36:4 |  | -7.37E-01 | 0.0414 | -0.085 | -0.0701 |  | -7.40E-01 | -0.051 | -0.0437 |  |  |  |  |  | |  | |  |  |  |  |
|  | PC ae C36:5 |  | -7.96E-01 | 0.0757 | -0.0738 | -0.0441 |  | -8.01E-01 | -0.0882 | -0.0411 |  |  |  |  |  | |  | |  |  |  |  |
|  | PC ae C38:0 |  | -8.18E-01 | 0.0683 | -0.0604 | 0.0499 |  | -8.02E-01 | -0.0579 | 0.021 |  |  |  |  |  | |  | |  |  |  |  |
|  | PC ae C38:5 |  | -8.06E-01 | 0.0475 | -0.0289 | -0.0529 |  | -8.16E-01 | -0.0596 | -0.0334 |  |  |  |  |  | |  | |  |  |  |  |
|  | PC ae C38:6 |  | -8.98E-01 | 0.1074 | -0.0715 | -0.0144 |  | -9.05E-01 | -0.1091 | -0.0253 |  |  |  |  |  | |  | |  |  |  |  |
|  | PC ae C40:2 |  | -7.35E-01 | 0.0896 | -0.0526 | 0.0792 |  | -7.16E-01 | -0.0827 | -0.0088 |  |  |  |  |  | |  | |  |  |  |  |
|  | PC ae C40:5 |  | -7.93E-01 | 0.0656 | -0.0494 | 0.0536 |  | -8.04E-01 | -0.072 | -0.075 |  |  |  |  |  | |  | |  |  |  |  |
|  | PC ae C40:6 |  | -8.91E-01 | 0.1334 | -0.0126 | 0.0822 |  | -9.00E-01 | -0.1343 | -0.0679 |  |  |  |  |  | |  | |  |  |  |  |
|  | PC ae C44:4 |  | -4.95E-01 | 0.0378 | -0.1027 | 0.0327 |  | -5.13E-01 | -0.0402 | 0.0105 |  |  |  |  |  | |  | |  |  |  |  |

# Table S3. Mean values (in units of standard deviation) of each individual feature with each of the omic profiles identified using Integrated/Quasi mediation.

|  |  |  | **Early Integration** | |  | **Intermediate Integration** | | | | | | | | | | | |  | | **Late Integration** | | | | | | | | | | | |  |
| --- | --- | --- | --- | --- | --- | --- | --- | --- | --- | --- | --- | --- | --- | --- | --- | --- | --- | --- | --- | --- | --- | --- | --- | --- | --- | --- | --- | --- | --- | --- | --- | --- |
| **Omics Layer** | **Feature Name** | **Associated Genes** | **Omic Profile 0** | **Omic Profile 1** |  | **Methyl-ome Profile 0** | | **Methyl-ome Profile 1** | | **mi-RNA Profile 0** | | **mi-RNA Profile 1** | | **Transcript-ome Profile 0** | | **Transcript-ome Profile 1** | |  | | **Methyl-ome Profile 0** | | **Methyl-ome Profile 1** | | **mi-RNA Profile 0** | | **mi-RNA Profile 1** | | **Transcript-ome Profile 0** | | **Transcript-ome Profile 1** | |  |
| *DNA Methylation* | *CpG Site* |  |  |  |  |  | |  | |  | |  | |  | |  | |  | |  | |  | |  | |  | |  | |  | |  |
|  | cg00659559 | *SCRN1* | - | - |  | 0.1 | | -1.3 | |  | |  | |  | |  | |  | | - | | - | |  | |  | |  | |  | |  |
|  | cg01119512 | *GRHL3;GRHL3-AS1* | -0.11 | 0.17 |  | -0.022 | | 0.29 | |  | |  | |  | |  | |  | | - | | - | |  | |  | |  | |  | |  |
|  | cg02053188 | *VTRNA1-3* | - | - |  | | 0.005 | | -0.065 | |  | |  | |  | |  | |  | | - | | - | |  | |  | |  | |  | |
|  | cg02096001 | *EIF1* | - | - |  | | -0.029 | | 0.37 | |  | |  | |  | |  | |  | | - | | - | |  | |  | |  | |  | |
|  | cg02116251 | - | 0.13 | -0.2 |  | | 0.055 | | -0.7 | |  | |  | |  | |  | |  | | - | | - | |  | |  | |  | |  | |
|  | cg04090745 | *HLA-DQB2* | - | - |  | | 0.072 | | -0.93 | |  | |  | |  | |  | |  | | - | | - | |  | |  | |  | |  | |
|  | cg05762852 | *HDGF;PRCC* | - | - |  | | 0.0017 | | -0.022 | |  | |  | |  | |  | |  | | - | | - | |  | |  | |  | |  | |
|  | cg05773599 | *WDR90* | - | - |  | | 0.054 | | -0.7 | |  | |  | |  | |  | |  | | - | | - | |  | |  | |  | |  | |
|  | cg05794325 | *BTF3L4;TXNDC12* | - | - |  | | 0.07 | | -0.9 | |  | |  | |  | |  | |  | | 0.059 | | -2.1 | |  | |  | |  | |  | |
|  | cg05898092 | *DUS1L* | - | - |  | | 0.01 | | -0.13 | |  | |  | |  | |  | |  | | - | | - | |  | |  | |  | |  | |
|  | cg07385577 | *AJ011932.1* | -0.089 | 0.13 |  | | -0.041 | | 0.53 | |  | |  | |  | |  | |  | | -0.049 | | 1.7 | |  | |  | |  | |  | |
|  | cg07948599 | *AL358472.7;CREB3L4;SLC39A1* | - | - |  | | 0.03 | | -0.39 | |  | |  | |  | |  | |  | | - | | - | |  | |  | |  | |  | |
|  | cg08111863 | *SHANK2* | - | - |  | | 0.02 | | -0.25 | |  | |  | |  | |  | |  | | - | | - | |  | |  | |  | |  | |
|  | cg08707475 | *AC025171.1;ZNF131* | - | - |  | | 0.036 | | -0.47 | |  | |  | |  | |  | |  | | - | | - | |  | |  | |  | |  | |
|  | cg13846866 | *EPM2AIP1;MLH1* | 0.039 | -0.059 |  | | 0.071 | | -0.92 | |  | |  | |  | |  | |  | | 0.058 | | -2 | |  | |  | |  | |  | |
|  | cg13971502 | *COL6A1* | - | - |  | | -0.022 | | 0.29 | |  | |  | |  | |  | |  | | - | | - | |  | |  | |  | |  | |
|  | cg14207210 | *PPM1E* | - | - |  | | 0.041 | | -0.53 | |  | |  | |  | |  | |  | | - | | - | |  | |  | |  | |  | |
|  | cg14659082 | *AC006538.2;SLC39A3* | - | - |  | | 0.023 | | -0.3 | |  | |  | |  | |  | |  | | - | | - | |  | |  | |  | |  | |
|  | cg16690859 | *FLJ40288;PLXNA4* | 0.058 | -0.088 |  | | 0.055 | | -0.71 | |  | |  | |  | |  | |  | | 0.036 | | -1.3 | |  | |  | |  | |  | |
|  | cg19965941 | *HSPD1;HSPE1-MOB4;MOB4* | -0.0017 | 0.0026 |  | | 0.041 | | -0.53 | |  | |  | |  | |  | |  | | - | | - | |  | |  | |  | |  | |
|  | cg20504025 | *SPCS2;XRRA1* | - | - |  | | 0.058 | | -0.75 | |  | |  | |  | |  | |  | | -0.0038 | | 0.13 | |  | |  | |  | |  | |
|  | cg21941251 | *TDRD5* | - | - |  | | 0.009 | | -0.12 | |  | |  | |  | |  | |  | | - | | - | |  | |  | |  | |  | |
|  | cg21972156 | *ZMIZ2* | 0.07 | -0.11 |  | | 0.072 | | -0.93 | |  | |  | |  | |  | |  | | 0.025 | | -0.88 | |  | |  | |  | |  | |
|  | cg24617363 | *NFYA;OARD1* | - | - |  | | -0.015 | | 0.2 | |  | |  | |  | |  | |  | | - | | - | |  | |  | |  | |  | |
|  | cg25627242 | *CILP2* | - | - |  | | -0.026 | | 0.34 | |  | |  | |  | |  | |  | | - | | - | |  | |  | |  | |  | |
|  | cg25823142 | *AJ011932.1* | - | - |  | | -0.023 | | 0.3 | |  | |  | |  | |  | |  | | - | | - | |  | |  | |  | |  | |
|  | cg26182263 | *SLC39A14* | - | - |  | | -0.0037 | | 0.048 | |  | |  | |  | |  | |  | | - | | - | |  | |  | |  | |  | |
|  | cg26853855 | *CSRNP3* | - | - |  | | -0.022 | | 0.28 | |  | |  | |  | |  | |  | | - | | - | |  | |  | |  | |  | |
| *Gene Transcription* | *Transcript Name* | *Associated Gene* |  |  |  | |  | |  | |  | |  | |  | |  | |  | |  | |  | |  | |  | |  | |  | |
|  | TC01006069.hg.1 | TC01006069.hg.1 (NONCODE) | -0.17 | 0.26 |  | |  | |  | |  | |  | | -0.099 | | 0.14 | |  | |  | |  | |  | |  | | - | | - | |
|  | TC02000627.hg.1 | SLC9A4 | - | - |  | |  | |  | |  | |  | | -0.058 | | 0.085 | |  | |  | |  | |  | |  | | - | | - | |
|  | TC02002300.hg.1 | RAB6C-AS1 | - | - |  | |  | |  | |  | |  | | -0.095 | | 0.14 | |  | |  | |  | |  | |  | | - | | - | |
|  | TC02004659.hg.1 | LOC100129029 | - | - |  | |  | |  | |  | |  | | -0.12 | | 0.18 | |  | |  | |  | |  | |  | | - | | - | |
|  | TC02004954.hg.1 | BRE | 0.12 | -0.18 |  | |  | |  | |  | |  | | 0.045 | | -0.065 | |  | |  | |  | |  | |  | | - | | - | |
|  | TC03001220.hg.1 | TC03001220.hg.1 (NONCODE) | 0.016 | -0.025 |  | |  | |  | |  | |  | | -0.0052 | | 0.0075 | |  | |  | |  | |  | |  | | - | | - | |
|  | TC04002114.hg.1 | TC04002114.hg.1 (NONCODE) | - | - |  | |  | |  | |  | |  | | 0.0021 | | -0.003 | |  | |  | |  | |  | |  | | - | | - | |
|  | TC04002369.hg.1 | TC04002369.hg.1 (NONCODE) | - | - |  | |  | |  | |  | |  | | -0.048 | | 0.07 | |  | |  | |  | |  | |  | | - | | - | |
|  | TC04002528.hg.1 | BEND4 | - | - |  | |  | |  | |  | |  | | -0.14 | | 0.21 | |  | |  | |  | |  | |  | | - | | - | |
|  | TC05001096.hg.1 | SLC9A3 | - | - |  | |  | |  | |  | |  | | -0.13 | | 0.19 | |  | |  | |  | |  | |  | | - | | - | |
|  | TC05001376.hg.1 | TC05001376.hg.1 (NONCODE) | - | - |  | |  | |  | |  | |  | | -0.025 | | 0.036 | |  | |  | |  | |  | |  | | - | | - | |
|  | TC06001648.hg.1 | TC06001648.hg.1 (NONCODE) | -0.048 | 0.073 |  | |  | |  | |  | |  | | -0.015 | | 0.023 | |  | |  | |  | |  | |  | | -0.017 | | 0.69 | |
|  | TC06001733.hg.1 | TC06001733.hg.1 (NONCODE) | - | - |  | |  | |  | |  | |  | | 0.079 | | -0.12 | |  | |  | |  | |  | |  | | - | | - | |
|  | TC06001809.hg.1 | ICK | - | - |  | |  | |  | |  | |  | | 0.12 | | -0.17 | |  | |  | |  | |  | |  | | - | | - | |
|  | TC07000748.hg.1 | HYALP1 | - | - |  | |  | |  | |  | |  | | -0.02 | | 0.029 | |  | |  | |  | |  | |  | | - | | - | |
|  | TC07002642.hg.1 | TC07002642.hg.1 (NONCODE) | - | - |  | |  | |  | |  | |  | | -0.039 | | 0.056 | |  | |  | |  | |  | |  | | -0.036 | | 1.5 | |
|  | TC08000864.hg.1 | TC08000864.hg.1 (NONCODE) | - | - |  | |  | |  | |  | |  | | -0.1 | | 0.15 | |  | |  | |  | |  | |  | | - | | - | |
|  | TC08001380.hg.1 | TC08001380.hg.1 (NONCODE) | -0.21 | 0.32 |  | |  | |  | |  | |  | | -0.049 | | 0.072 | |  | |  | |  | |  | |  | | -0.025 | | 1 | |
|  | TC09001768.hg.1 | C9orf173-AS1 | - | - |  | |  | |  | |  | |  | | -0.0099 | | 0.014 | |  | |  | |  | |  | |  | | - | | - | |
|  | TC09002871.hg.1 | C9orf173-AS1 | - | - |  | |  | |  | |  | |  | | -0.016 | | 0.023 | |  | |  | |  | |  | |  | | - | | - | |
|  | TC10000010.hg.1 | WDR37 | - | - |  | |  | |  | |  | |  | | 0.13 | | -0.19 | |  | |  | |  | |  | |  | | 0.033 | | -1.3 | |
|  | TC10000169.hg.1 | KIAA1217 | - | - |  | |  | |  | |  | |  | | -0.11 | | 0.16 | |  | |  | |  | |  | |  | | - | | - | |
|  | TC10002518.hg.1 | LINC00614 | - | - |  | |  | |  | |  | |  | | -0.073 | | 0.11 | |  | |  | |  | |  | |  | | - | | - | |
|  | TC12001689.hg.1 | GRIP1 | - | - |  | |  | |  | |  | |  | | -0.17 | | 0.25 | |  | |  | |  | |  | |  | | - | | - | |
|  | TC17001096.hg.1 | LOC284023 | - | - |  | |  | |  | |  | |  | | -0.13 | | 0.2 | |  | |  | |  | |  | |  | | - | | - | |
|  | TC19000509.hg.1 | ZNF568 | - | - |  | |  | |  | |  | |  | | 0.03 | | -0.043 | |  | |  | |  | |  | |  | | - | | - | |
|  | TC20001151.hg.1 | TC20001151.hg.1 (NONCODE) | - | - |  | |  | |  | |  | |  | | -0.068 | | 0.099 | |  | |  | |  | |  | |  | | - | | - | |
|  | TC22000983.hg.1 | TC22000983.hg.1 (NONCODE) | -0.036 | 0.054 |  | |  | |  | |  | |  | | 0.19 | | -0.27 | |  | |  | |  | |  | |  | | - | | - | |
| *miRNA* | *miRNA Name* | *miRNA Name* |  |  |  | |  | |  | |  | |  | |  | |  | |  | |  | |  | |  | |  | |  | |  | |
|  | hsa-miR-101-3p | hsa-miR-101-3p | - | - |  | |  | |  | | 0.65 | | -0.63 | |  | |  | |  | |  | |  | | 0.25 | | -0.25 | |  | |  | |
|  | hsa-miR-125a-5p | hsa-miR-125a-5p | - | - |  | |  | |  | | 0.19 | | -0.19 | |  | |  | |  | |  | |  | | - | | - | |  | |  | |
|  | hsa-miR-125b-1-3p | hsa-miR-125b-1-3p | - | - |  | |  | |  | | 0.091 | | -0.088 | |  | |  | |  | |  | |  | | - | | - | |  | |  | |
|  | hsa-miR-127-3p | hsa-miR-127-3p | - | - |  | |  | |  | | -0.089 | | 0.086 | |  | |  | |  | |  | |  | | -0.017 | | 0.017 | |  | |  | |
|  | hsa-miR-140-5p | hsa-miR-140-5p | - | - |  | |  | |  | | 0.53 | | -0.51 | |  | |  | |  | |  | |  | | -0.0031 | | 0.0031 | |  | |  | |
|  | hsa-miR-142-3p | hsa-miR-142-3p | - | - |  | |  | |  | | 0.58 | | -0.56 | |  | |  | |  | |  | |  | | - | | - | |  | |  | |
|  | hsa-miR-144-5p | hsa-miR-144-5p | - | - |  | |  | |  | | 0.48 | | -0.47 | |  | |  | |  | |  | |  | | - | | - | |  | |  | |
|  | hsa-miR-19a-3p | hsa-miR-19a-3p | - | - |  | |  | |  | | 0.56 | | -0.55 | |  | |  | |  | |  | |  | | - | | - | |  | |  | |
|  | hsa-miR-19b-3p | hsa-miR-19b-3p | - | - |  | |  | |  | | 0.64 | | -0.62 | |  | |  | |  | |  | |  | | 0.14 | | -0.14 | |  | |  | |
|  | hsa-miR-21-5p | hsa-miR-21-5p | - | - |  | |  | |  | | 0.58 | | -0.56 | |  | |  | |  | |  | |  | | 0.23 | | -0.24 | |  | |  | |
|  | hsa-miR-2110 | hsa-miR-2110 | - | - |  | |  | |  | | 0.3 | | -0.29 | |  | |  | |  | |  | |  | | - | | - | |  | |  | |
|  | hsa-miR-215-5p | hsa-miR-215-5p | - | - |  | |  | |  | | 0.55 | | -0.53 | |  | |  | |  | |  | |  | | 0.19 | | -0.19 | |  | |  | |
|  | hsa-miR-27a-3p | hsa-miR-27a-3p | - | - |  | |  | |  | | 0.52 | | -0.51 | |  | |  | |  | |  | |  | | - | | - | |  | |  | |
|  | hsa-miR-29c-3p | hsa-miR-29c-3p | - | - |  | |  | |  | | 0.55 | | -0.53 | |  | |  | |  | |  | |  | | - | | - | |  | |  | |
|  | hsa-miR-30b-5p | hsa-miR-30b-5p | - | - |  | |  | |  | | 0.55 | | -0.53 | |  | |  | |  | |  | |  | | 0.24 | | -0.25 | |  | |  | |
|  | hsa-miR-331-3p | hsa-miR-331-3p | - | - |  | |  | |  | | 0.47 | | -0.46 | |  | |  | |  | |  | |  | | - | | - | |  | |  | |
|  | hsa-miR-3614-5p | hsa-miR-3614-5p | - | - |  | |  | |  | | -0.031 | | 0.03 | |  | |  | |  | |  | |  | | - | | - | |  | |  | |
|  | hsa-miR-365a-3p | hsa-miR-365a-3p | - | - |  | |  | |  | | 0.32 | | -0.31 | |  | |  | |  | |  | |  | | -0.064 | | 0.065 | |  | |  | |
|  | hsa-miR-365b-5p | hsa-miR-365b-5p | - | - |  | |  | |  | | 0.024 | | -0.024 | |  | |  | |  | |  | |  | | - | | - | |  | |  | |
|  | hsa-miR-3685 | hsa-miR-3685 | - | - |  | |  | |  | | -0.055 | | 0.054 | |  | |  | |  | |  | |  | | -0.27 | | 0.27 | |  | |  | |
|  | hsa-miR-371a-3p | hsa-miR-371a-3p | 0.13 | -0.2 |  | |  | |  | | -0.15 | | 0.14 | |  | |  | |  | |  | |  | | -0.39 | | 0.39 | |  | |  | |
|  | hsa-miR-4743-5p | hsa-miR-4743-5p | - | - |  | |  | |  | | 0.05 | | -0.048 | |  | |  | |  | |  | |  | | 0.26 | | -0.27 | |  | |  | |
|  | hsa-miR-486-5p | hsa-miR-486-5p | - | - |  | |  | |  | | 0.32 | | -0.31 | |  | |  | |  | |  | |  | | - | | - | |  | |  | |
|  | hsa-miR-520b | hsa-miR-520b | - | - |  | |  | |  | | -0.12 | | 0.12 | |  | |  | |  | |  | |  | | -0.21 | | 0.2 | |  | |  | |
|  | hsa-miR-551b-3p | hsa-miR-551b-3p | 0.15 | -0.23 |  | |  | |  | | -0.1 | | 0.097 | |  | |  | |  | |  | |  | | -0.33 | | 0.33 | |  | |  | |
|  | hsa-miR-6073 | hsa-miR-6073 | - | - |  | |  | |  | | 0.063 | | -0.061 | |  | |  | |  | |  | |  | | - | | - | |  | |  | |
|  | hsa-miR-628-3p | hsa-miR-628-3p | - | - |  | |  | |  | | 0.15 | | -0.14 | |  | |  | |  | |  | |  | | - | | - | |  | |  | |
|  | hsa-miR-6876-5p | hsa-miR-6876-5p | 0.019 | -0.029 |  | |  | |  | | 0.12 | | -0.11 | |  | |  | |  | |  | |  | | - | | - | |  | |  | |
